# Effect of modifiable risk factors on cardiovascular and cancer mortality across European and Asian regions: a multicohort study

**DOI:** 10.1016/j.lanepe.2026.101918

**Published:** 2026-11-09

**Authors:** A Eurasian Cardiovascular and Cancer Risk Collaboration, Mahshid Ghasempour Moghaddam, Elisa Großmann, Linlin Guo, Davood Khalili, Marie Neumann, Tetsu Watanabe, Thiess Lorenz, Bamba Gaye, Lubna A. Alnasser, Philippe Amouyel, Fereidoun Azizi, Dirk De Bacquer, Stephan J.L. Bakker, Martin Bobak, Paolo Boffetta, Hermann Brenner, Chean Lin Chong, Simona Costanzo, Giovanni de Gaetano, Robin P.F. Dullaart, Johann A.C. Edjimbi, Gunnar Engström, Victoria Gauthier, Yariv Gerber, Uri Goldbourt, Stefan Groß, Farzad Hadaegh, Kristian Hveem, Licia Iacoviello, Manami Inoue, Till Ittermann, Mohamed B. Jalloh, Gorm Boje Jensen, Anna Kontsevaya, Magnus Nakrem Lyngbakken, Darryl P. Leong, Pasquale Maffia, Reza Malekzadeh, Olle Melander, Hossein Molavi Vardanjani, Julia Munzinger, Toshiharu Ninomiya, Torbjørn Omland, Sok King Ong, Yoichiro Otaki, Andrzej Pająk, Arokiasamy Perianayagam, Peter M. Nilsson, Hossein Poustchi, Eva Prescott, Satoko Sakata, Ben Schöttker, Sadaf G. Sepanlou, Sanjib Kumar Sharma, Raphael Twerenbold, Masafumi Watanabe, Stefan Blankenberg, Francisco M. Ojeda, Christina Magnussen

**Affiliations:** a Department of Cardiology, University Heart and Vascular Center Hamburg, University Medical Center Hamburg-Eppendorf, Hamburg, Germany; b German Center for Cardiovascular Research (DZHK), Partner Site North, Hamburg, Germany; c Center for Population Health Innovation, University Heart and Vascular Center Hamburg, University Medical Center Hamburg-Eppendorf, Hamburg, Germany; d Alliance for Medical Research in Africa, Dakar, Senegal; e Nuffield Department of Medicine, University of Oxford, Oxford, UK; f PARCC, Université Paris Cité, INSERM U970, Paris, France; g Department of Population Health, King Abdullah International Medical Research Center, King Saud Bin Abdulaziz University for Health Sciences, Riyadh, Saudi Arabia; h University of Lille, Inserm, Lille University Hospital, Institut Pasteur de Lille, U1167 – RID-AGE – Risk Factors and Molecular Determinants of Aging-Related Diseases, Lille, France; i Endocrine Research Center, Research Institute for Endocrine Sciences, Shahid Beheshti University of Medical Sciences, Tehran, Iran; j Department of Public Health and Primary Care, Ghent University, Ghent, Belgium; k Department of Internal Medicine, University Medical Center Groningen, University of Groningen, Groningen, the Netherlands; l Institute of Epidemiology and Health Care, University College London, London, UK; m RECETOX, Masaryk University, Brno, Czech Republic; n Department of Family, Population, and Preventive Medicine, Stony Brook University, Stony Brook, NY, USA; o Stony Brook Cancer Center, Stony Brook University, Stony Brook, NY, USA; p Department of Medical and Surgical Sciences, University of Bologna, Bologna, Italy; q Cancer Prevention Graduate School, German Cancer Research Center (DKFZ), Heidelberg, Germany; r Network Aging Research, Heidelberg University, Heidelberg, Germany; s PAPRSB Institute of Health Sciences, Universiti Brunei Darussalam, Bandar Seri Begawan, Brunei Darussalam; t Research Unit of Epidemiology and Prevention, IRCCS Neuromed, Pozzilli, Italy; u Department of Medicine and Surgery, Research Center in Epidemiology and Preventive Medicine, University of Insubria, Varese, Italy; v Department of Clinical Sciences in Malmö, Lund University, Lund, Sweden; w Department of Epidemiology and Preventive Medicine, School of Public Health, Gray Faculty of Medical and Health Sciences, Tel Aviv University, Israel; x German Center for Cardiovascular Research (DZHK), DZHK Partnersite North, Greifswald, Germany; y Internal Medicine B, Department of Cardiology, University Medicine Greifswald, Greifswald, Germany; z Prevention of Metabolic Disorders Research Center, Research Institute for Metabolic and Obesity Disorders, Research Institute for Endocrine Sciences, Shahid Beheshti University of Medical Sciences, Tehran, Iran; aa HUNT Center for Molecular and Clinical Epidemiology, Department of Public Health and Nursing, Norwegian University of Science and Technology, Trondheim, Norway; ab Department of Research and Education, St. Olav’s Hospital, Trondheim, Norway; ac Department of Medicine and Surgery, LUM University, Casamassima, Italy; ad Institute for Cancer Control, National Cancer Center, Tokyo, Japan; ae Department of Cardiology, Bispebjerg Hospital, Copenhagen, Denmark; af National Medical Research Center for Therapy and Preventive Medicine, Moscow, Russia; ag Department of Cardiology, Division of Medicine, Akershus University Hospital, Lørenskog, Norway; ah K.G. Jebsen Center for Cardiac Biomarkers, Institute of Clinical Medicine, University of Oslo, Oslo, Norway; ai McMaster University, Hamilton, Canada; aj School of Infection & Immunity, College of Medicine and Veterinary Sciences, University of Glasgow, Glasgow, UK; ak Department of Pharmacy, School of Medicine and Surgery, University of Naples Federico II, Naples, Italy; al Digestive Oncology Research Center, Digestive Disease Research Institute, Shariati Hospital, Tehran University of Medical Sciences, Tehran, Iran; am Department of Clinical Sciences, Lund University, Malmö, Sweden; an MPH Department, School of Medicine, Research Center for Traditional Medicine and History of Medicine, Shiraz University of Medical Sciences, Shiraz, Iran; ao Department of Epidemiology and Public Health, Graduate School of Medical Sciences, Kyushu University, Japan; ap Department of Cardiology, Pulmonology, and Nephrology, Yamagata University School of Medi-cine, Yamagata, Japan; aq Department of Epidemiology and Population Studies, Institute of Public Health, Faculty of Health Sciences, Jagiellonian University Medical College, Krakow, Poland; ar Social and Economic Survey Research Institute, Qatar University, Doha, Qatar; as Liver and Pancreatobiliary Diseases Research Center, Digestive Disease Research Institute, Tehran University of Medical Sciences, Tehran, Iran; at Division of Clinical Epidemiology of Early Cancer Detection, German Cancer Research Center (DKFZ), Heidelberg, Germany; au Department of Internal Medicine, B.P. Koirala Institute of Health Sciences, Dharan, Nepal; av Yamagata University Faculty of Medicine, Yamagata, Japan

**Keywords:** Cardiovascular disease epidemiology, Cancer epidemiology, Modifiable risk factors, Public health, Noncommunicable diseases

## Abstract

**Background:**

Cardiovascular disease and cancer are the leading causes of death worldwide, and a substantial proportion of mortality is attributable to modifiable risk factors. This study aimed to assess the associations of modifiable risk factors with cardiovascular and cancer mortality across European and Asian populations.

**Methods:**

We analysed harmonized data from 798,867 adults in 25 cohorts across Europe, Western Asia and North Africa, and Central, East and South Asia, using two-stage random-effects individual-participant-data meta-analysis Cox and Weibull models to estimate hazard ratios and population-attributable fractions (PAFs). PAFs were estimated for each individual risk factor and jointly for all risk factors. Risk factors included body mass index, systolic blood pressure, non–HDL cholesterol, current smoking, and diabetes. The main outcomes were 10-year risks for cardiovascular and cancer mortality.

**Findings:**

During a median follow-up of 8.2 years, 12,429 cardiovascular and 19,674 cancer deaths occurred. Deaths per 1000 person-years were higher among men for cardiovascular mortality (2.4, 95% CI 2.3–2.4) than women (1.4, 95% CI 1.4–1.5), as was cancer mortality (for men 3.6, 95% CI 3.5–3.7; for women 2.3, 95% CI 2.4–2.5). Joint PAFs were larger for cardiovascular than cancer mortality for women (52.3%, 95% CI 41.9–62.7, vs. 16.3%, 6.0–26.6) and men (44.4%, 95% CI 33.8–54.9, vs. 29.3%, 20.0–38.7) across regions. Systolic blood pressure and diabetes contributed most to cardiovascular mortality, while smoking was the main driver of cancer mortality, particularly in men. For cardiovascular mortality, joint PAFs were lowest in European men (37.5%, 95% CI 24.1–50.9) and highest in Western Asian and North African women (61.0%, 95% CI 51.7–70.3). For cancer mortality, joint PAFs were lowest in European women (14.7%, 95% CI 3.8–25.6) and highest in Central, East and South Asian men (34.5%, 95% CI 25.7–43.3).

**Interpretation:**

Modifiable risk factors accounted for a larger proportion of cardiovascular than cancer deaths, with variations by sex and region. Prioritizing interventions for blood pressure, diabetes, and tobacco cessation could reduce mortality and improve region-specific prevention.

**Funding:**

None.

**Research in context**

**Evidence before this study**

We searched PubMed and Web of Science for studies published between 1 January 2001 and 31 January 2026, without language restrictions. Search terms combined terms for cardiovascular mortality, cancer mortality, smoking, systolic blood pressure, hypertension, diabetes, body-mass index, obesity, non-HDL cholesterol, dyslipidaemia, and educational attainment, including relevant synonyms and truncations. We considered observational population-based studies, systematic reviews, and meta-analyses examining associations between these risk factors or educational attainment and cardiovascular and cancer mortality. The available evidence was assessed qualitatively, considering study design, population coverage, consistency of findings, and potential sources of bias. Large international collaborations, including the Global Cardiovascular Risk Consortium, have shown that a substantial proportion of cardiovascular disease incidence and mortality is attributable to a limited number of modifiable risk factors, with marked variation by sex and geographic region. Previous analyses have mainly focused on cardiovascular outcomes and all-cause mortality. Evidence on the joint impact of established cardiovascular risk factors on cancer mortality using harmonised individual-level data across multiple regions remains limited. Although several risk factors such as smoking, obesity, hypertension, and diabetes are known to contribute to both cardiovascular disease and cancer, their relative contributions to cause-specific mortality and their preventable burden across regions have not been comprehensively quantified.

**Added value of this study**

Using harmonised individual-level data from 798,867 adults across 25 prospective cohorts in Europe, the Middle East and North Africa, and Asia, this study provides a large multi-cohort, multi-region assessment of shared modifiable risk factors for cardiovascular and cancer mortality. We quantify sex-specific population-attributable fractions for 10-year cardiovascular and cancer mortality, and 20-year mortality risks at the index age of 50 to assess premature mortality. The findings show that the preventable burden attributable to these risk factors is substantially larger for cardiovascular mortality than for cancer mortality, with important variation by sex and region. In addition, educational attainment emerged as an additional contextual modifier of these associations, suggesting that differences in risk factor control, healthcare access, and treatment pathways contribute to the observed heterogeneity in preventable mortality across regions.

**Implications of all the available evidence**

Together with previous studies, these findings support the prioritisation of integrated prevention strategies targeting shared upstream risk factors, particularly elevated blood pressure, diabetes, and tobacco use, to reduce overall and premature mortality from both cardiovascular disease and cancer. The results also highlight the importance of addressing socioeconomic inequalities, in particular disparities in educational attainment, which likely influence risk factor exposure, access to preventive care, and long-term disease management. Region-specific prevention policies, strengthened primary care systems, and equitable access to risk factor screening and treatment could yield substantial public health benefits across diverse settings.

## Introduction

Cardiovascular disease and cancer are the leading global causes of death, with a large proportion attributable to potentially modifiable risk factors.^1^, ^2^ Analyses from the Global Cardiovascular Risk Consortium indicate that five classic modifiable risk factors–obesity, hypertension, dyslipidemia, current smoking, and diabetes–account for a large proportion of incident cardiovascular disease and death.^3^ However, the prevalence and clustering of these risk factors, as well as access to prevention and treatment, vary widely by geography and socioeconomic context, including Europe, Western Asia and North Africa, and Central, East and South Asia.^4^, ^5^ While cancer and cardiovascular disease are major contributors to overall mortality across Europe, Western Asia and North Africa, and Central, East and South Asia, their relative burden differ substantially, reflecting the diverse demographic transitions, risk factor profiles, and health system characteristics of these regions. These regions also differ in guideline recommendations for modifiable risk factors, allowing our analyses to inform both regional alignment and context-specific prevention strategies.

In addition, cancer and cardiovascular outcomes are increasingly recognized as interrelated chronic diseases that share common modifiable risk factors and mortality pathways.^6^ Due to this interdependence, the evaluation of established modifiable cardiovascular risk factors on both cardiovascular and cancer outcomes may inform integrated prevention strategies. Moreover, sex-specific differences in risk factor distribution, biological and hormonal influences, and preventive care may contribute to heterogeneity in these associations, warranting sex-stratified analyses.

Therefore, this study aimed to quantify and compare the associations of potentially modifiable risk factors with cardiovascular and cancer mortality in Europe, Western Asia and Central, East and South Asia and to assess variation by sex and geographic region.

## Methods

### Study population

Harmonized individual-level data were analysed from 798,867 participants (aged ≥ 18 years) enrolled in 25 prospective cohort studies from three geographic regions–Europe, Western Asia and North Africa, and Central, East and South Asia–participating in the *Eurasian Cardiovascular and Cancer Risk Collaboration*. Regions were informed by World Bank classifications^7^ and adapted based on data availability and geographic distribution. Individuals with prevalent cardiovascular disease (history of stroke, unstable angina pectoris, coronary heart disease or cardiac revascularization) or cancer at baseline or missing follow-up information on cardiovascular death (death from stroke, coronary heart disease or sudden cardiac arrest) or cancer death were excluded from regression models and population-attributable fraction (PAF) calculations. Participating cohorts were independent population-based prospective studies conducted in individual countries and did not follow a uniform study design. Cohorts were selected on the basis of availability of the key risk factors analysed in this study (body mass index, systolic blood pressure, non–HDL cholesterol, current smoking, and diabetes) and availability of follow-up data for cardiovascular and cancer mortality. Additional details on cohort characteristics, recruitment procedures, harmonization, and selection criteria are provided in the Supplementary methods.

In addition to prospective cohort studies, cross-sectional population-based health examination survey data were used to contextualize contemporary regional risk-factor distributions.

### Data harmonization

All data were harmonized using the variable definitions of the MORGAM (MONICA, Risk, Genetics, Archiving, and Monograph) cohort consortium.^8^ Harmonization included standardized recoding of demographic variables, medical history, risk factors, medication use, and mortality outcomes across cohorts with differing study designs and data collection procedures. The harmonization process is described in the Supplementary methods. Further details on how each variable was defined and coded, can be found in the Supplementary appendix.

### Risk factors and outcomes

Risk factors were assessed once at study entry. On-site examinations ascertained diabetes status and current smoking (any regular or occasional tobacco use, excluding e-cigarettes), recorded educational attainment, and measured body mass index (BMI), systolic blood pressure (SBP), and non–high-density lipoprotein cholesterol (non–HDL-C). Prevalent cardiovascular disease was defined as a history of unstable angina, coronary heart disease, stroke or cardiac revascularization. Prevalent cancer was defined as a history of any cancer excluding melanoma skin cancer.

Continuous risk factors were categorized for more detailed PAF estimates. The categories for BMI, systolic blood pressure, and non-HDL-C were primarily based on standard classifications provided by the World Health Organization. Specifically, BMI was categorized as: <20 kg/m^2^ (underweight), 20 ≤ BMI <25 kg/m^2^ (normal weight), 25 ≤ BMI <30 kg/m^2^ (overweight), and BMI ≥30 kg/m^2^ (obese); SBP was categorized as: 120 ≤ SBP<140 mmHg (normal to elevated), 140 ≤ SBP <160 mmHg (grade 1 hypertension), and systolic blood pressure ≥160 mmHg (grade 2-3 hypertension); and non-HDL-C was categorized as: 3 ≤ non-HDL-C < 4 mmol/L (desirable), 4 ≤ non-HDL-C < 5 mmol/L (borderline high), and non-HDL-C ≥ 5 mmol/L (high). Education was categorized into cohort-specific tertiles of completed years of education.^9^

Outcomes were defined by ICD codes for coronary heart disease, sudden cardiac arrest, stroke, and cancer, and were ascertained from death certificates or linkage with registries or medical records. Premature mortality was defined as deaths before 70 years of age.^10^ Detailed definitions and methods of obtaining and validating mortality data are provided in the Supplementary appendix.

### Statistical analyses

Missing data was addressed using multiple imputation with chained equations.^11^ Analyses were performed separately for women and men. 10-year mortality risks and event rates per 1000 person-years were estimated. Cumulative incidence curves for cardiovascular and cancer mortality were estimated with age as the time scale and with time since study entry. For each outcome, death from other causes (non-cardiovascular death for cardiovascular mortality; non-cancer death for cancer mortality) was treated as a competing event, and cumulative incidence was estimated with the Aalen-Johansen estimator.^12^

Associations between risk factors and outcomes were calculated by performing a two-stage, random-effects multivariate individual-participant-data meta-analysis.^13^ Proportional hazard models were used, Cox models for association analyses and Weibull models for the PAF analyses. The model parameters were pooled by region and overall using multivariate random-effects meta-analysis. All models included age as the time scale, and the following exposures: BMI, SBP, non-HDL-C, current smoking, and diabetes, and use of antihypertensive medication. Three different sets of Cox models were computed: 1) all exposures entered linearly; 2) continuous exposures (BMI, SBP, and non-HDL-C) were modelled using restricted cubic splines (four knots, identical across all studies); and 3) models allowing exposures to vary with age (time scale) thereby relaxing the proportional hazards assumption and evaluating potential time-varying associations between risk factors and cardiovascular and cancer mortality. For the association and PAF analyses, we performed a 1-year landmark analysis by excluding individuals who died within the first year of follow-up and restricting the analysis to those alive at one year. This approach was intended to reduce potential reverse causation, whereby baseline risk factors might be influenced by undiagnosed disease present at study entry, leading to deaths shortly after baseline and potentially biasing the observed associations. The choice of a 1-year period reflects a pragmatic balance between minimising this bias and preserving sufficient follow-up time and statistical power. In a secondary analysis, completed years of education were added to the exposure set.

PAFs for the 10-year risks of cardiovascular and cancer mortality were estimated using the methods from Laaksonen and colleagues.^14^ This approach allows the estimation of PAFs for combinations of risk factors. Weibull proportional hazard models were used to estimate the 10-year event probabilities required for the PAF estimation. PAF estimation was based on the time-to-event models derived on the cohort studies. Health examination survey data were not used in the estimation of these models, but served to provide contemporary regional population-level risk-factor distributions. PAFs for premature death^10^ were calculated estimating 20-year incidence of cardiovascular and cancer mortality for individuals aged 50 years at baseline. All statistical methods were implemented in R statistical language, version 4.3.3.^15^

### Healthcare expenditure

To assess the potential for translating PAF results into real-world settings, current health expenditure per capita was obtained from WHO data^16^ for the survey year of each health examination survey used. Regional current health expenditure estimates were population-weighted using country-level population data from the World Bank.^17^ These estimates were used to provide contextual information on regional health system resources rather than individual-level expenditure.

### Sensitivity analyses

Due to the differences in cardiovascular and cancer mortality within the UK Biobank, we repeated incidence and PAF analyses after excluding the UK Biobank. Additionally, we performed Cox regression analyses also for non-cardiovascular and non-cancer mortality. To evaluate whether simultaneous adjustment for multiple risk factors influenced the reported associations, additional Cox regression analyses were performed for each exposure–outcome association, adjusting for relevant confounders. For diabetes, analyses were performed both with and without adjustment for BMI to evaluate the influence of adiposity on the estimated associations. To adjust for potential birth cohort effects, linear and non-linear Cox regression were calculated adjusting for the cohorts’ examination year. Furthermore, we fitted linear Cox regression models restricted to cohorts recruited after 1988 to assess potential temporal heterogeneity across cohorts.

### Role of the funding source

This study received no specific funding. No funder had any role in the study design, data collection, analysis or interpretation, writing of the manuscript, or the decision to submit the manuscript for publication.

## Results

### Risk factor differences by sex and region

In analysis of 798,867 individuals from 25 prospective cohorts in Europe, Western Asia and North Africa, and Central, East and South Asia, 53.7% were women and the median age at baseline was 56.8 years (interquartile range, 48.2–63.5) (Table 1). Risk factor profiles differed across regions: individuals in Central, East and South Asia had lower median BMI and non-HDL-C levels but the highest prevalence of current smoking (22.7%); participants in Europe had the highest prevalence of elevated SBP; and participants in Western Asia and North Africa had the highest prevalence of diabetes (10.0%). Supplementary Table S5 displays an age- and sex-standardized distribution of baseline characteristics. Baseline characteristics of individuals from the health examination surveys are shown in Supplementary Table S6.

**Table 1:** Cohorts' baseline characteristics according to geographic region.

	Overall	Europe	Western Asia and North Africa	Central, East and South Asia
N participants	798,867	703,470	42,789	52,608
N cohorts	25	17	4	4
Survey year, range	1963–2019; NAs:0	1976–2015; NAs:0	1963–2015; NAs:0	1988–2019; NAs:0
Female No. (%)	429,075 (53.7); NAs:0	379,777 (54.0); NAs:0	17,966 (42.0); NAs:0	31,332 (59.6); NAs:0
Examination age (years)	56.8 (48.2, 63.5); NAs:0	57.3 (49.1, 63.7); NAs:0	50.0 (43.0, 57.8); NAs:0	54.0 (38.4, 65.0); NAs:0
Body mass index (kg/m^2^)	26.3 (23.7, 29.4); NAs:13,183	26.6 (24.0, 29.7); NAs:7180	26.1 (23.3, 29.1); NAs:472	23.0 (20.7, 25.6); NAs:5531
Non-HDL cholesterol (mmol/L)	4.2 (3.4, 4.9); NAs:150,226	4.2 (3.5, 5.0); NAs:116,762	3.9 (3.2, 4.6); NAs:3851	3.6 (3.0, 4.2); NAs:29,613
Systolic blood pressure (mmHg)	134.5 (122.0, 148.5); NAs:6371	136.0 (124.0, 150.0); NAs:5403	120.0 (110.0, 135.0); NAs:309	120.0 (110.0, 136.0); NAs:659
Diastolic blood pressure (mmHg)	81.0 (74.5, 89.0); NAs:6408	81.5 (75.0, 89.0); NAs:5437	80.0 (70.0, 85.0); NAs:312	80.0 (70.0, 86.0); NAs:659
Current smoking No. (%)	13,4054 (16.9); NAs:7009	113,410 (16.3); NAs:5868	8882 (20.9); NAs:281	11,762 (22.7); NAs:860
Diabetes No. (%)	45,626 (5.8); NAs:15,279	37,294 (5.4); NAs:9287	4212 (10.0); NAs:709	4120 (8.7); NAs:5283
Years of education (years)	14.0 (10.0, 20.0); NAs:147,174	15.0 (10.0, 20.0); NAs:82,631	12.0 (12.0, 14.0); NAs:30,351	10.0 (0.0, 14.0); NAs:34,192
Antihypertensive medication No. (%)	153,121 (20.2); NAs:42,482	140,635 (21.2); NAs:40,422	5154 (12.3); NAs:822	7332 (14.3); NAs:1238
Lipid-lowering medication No. (%)	97,993 (14.6); NAs:127,844	94,251 (15.6); NAs:99,757	1371 (3.2); NAs:271	2371 (9.6); NAs:27,816
History of CVD No. (%)	36,806 (4.6); NAs:0	32,429 (4.6); NAs:0	3033 (7.1); NAs:0	1344 (2.6); NAs:0
History of any cancer No. (%)	68,202 (8.5); NAs:0	67,885 (9.7); NAs:0	239 (0.6); NAs:0	78 (0.1); NAs:0

Continuous variables are presented as medians with interquartile ranges (25th and 75th percentiles), except for 'survey year', for which the minimum and maximum values are reported. Binary variables are summarized using relative frequencies. Percentages and quartiles are calculated based on the number of available cases for each variable. Missing values are listed beneath each value. ‘Examination age’ denotes information about the age of participants at the beginning of the study. NA, Not available; non-HDL, non-high-density lipoprotein.

### Cardiovascular and cancer mortality

A total of 12,429 cardiovascular deaths and 19,674 deaths from cancer were observed. Age-standardized 10-year mortality rates differed by sex and geographic region (Table 2). Cardiovascular and cancer mortality was consistently higher among men than women. Among women, the overall 10-year risk of cardiovascular mortality was 0.9% (95% confidence interval [CI], 0.9–1.0), and the risk of cancer mortality was 1.9% (95% CI 1.8–1.9). Cardiovascular mortality was highest in Western Asia and North Africa (2.1%) and lowest in Central, East and South Asia (0.9%), whereas cancer mortality was highest in Europe (1.9%) and lowest again in Central, East and South Asia (1.2%). Among men, the overall 10-year risk of cardiovascular mortality was 1.8% (95% CI 1.8–1.9), and the risk of cancer mortality was 3.1% (95% CI 3.0–3.2). Cardiovascular mortality risk was highest in Western Asia and North Africa (5.1%) and lowest in Central, East and South Asia (1.3%), while cancer mortality was highest in Central, East and South Asia (3.2%) and lowest in Western Asia and North Africa (2.9%).

**Table 2:** Age-standardized 10-year incidences (%) and incidence rates per 1000 person-years for cardiovascular and cancer mortality according to sex and region.

	Cardiovascular mortality			Cancer mortality		
	No. of events	10-year incidence % (95% CI)	Per 1000 person-years (95% CI)	No. of events	10-year incidence % (95% CI)	Per 1000 person-years (95% CI)
Women						
Overall	4927	0.9 (0.9, 1.0)	1.4 (1.4, 1.5)	8458	1.9 (1.8, 1.9)	2.3 (2.3, 2.4)
Europe	4600	0.9 (0.8, 0.9)	1.4 (1.4, 1.5)	8129	1.9 (1.8, 2.0)	2.4 (2.4, 2.5)
Western Asia and North Africa	208	2.1 (1.7, 2.4)	2.2 (1.9, 2.5)	158	1.5 (1.1, 1.8)	1.6 (1.3, 1.9)
Central, East and South Asia	119	0.9 (0.5, 1.3)	0.7 (0.6, 0.9)	171	1.2 (0.8, 1.7)	1.3 (1.1, 1.6)
Men						
Overall	7443	1.8 (1.8, 1.9)	2.4 (2.4, 2.5)	11216	3.1 (3.0, 3.2)	3.6 (3.5, 3.7)
Europe	5712	1.6 (1.6, 1.7)	2.1 (2.1, 2.2)	10026	3.1 (3.0, 3.2)	3.6 (3.5, 3.7)
Western Asia and North Africa	1597	5.1 (4.7, 5.6)	6.6 (6.2, 7.0)	894	2.9 (2.5, 3.3)	3.8 (3.5, 4.1)
Central, East and South Asia	134	1.3 (0.8, 1.8)	1.3 (1.1, 1.6)	296	3.2 (2.4, 4.1)	3.3 (2.9, 3.8)

CI, confidence interval.

The overall and cohort-specific follow-up durations and event numbers are shown in Supplementary Table S7 for cardiovascular and cancer mortality.

Cumulative incidence curves using age as the time scale showed steep increases in both cardiovascular and cancer mortality with advancing age, with higher mortality at younger ages in men than in women (Supplementary Fig. S4a). Cumulative incidence curves using time since enrollment as the time scale increased more sharply after about 20 years of follow-up in women and 15 years in men (Supplementary Fig. S4b).

Exclusion of the UK Biobank had minimal effects and did not alter overall trajectories (Supplementary Fig. S5).

### Hazard ratios for risk factors and outcomes

The largest sex-specific differences were observed for SBP and non-HDL-C: SBP was more strongly associated with cancer mortality in men, whereas non-HDL-C showed a less inverse relationship with cancer mortality in women (Fig. 1).

**Fig. 1:**
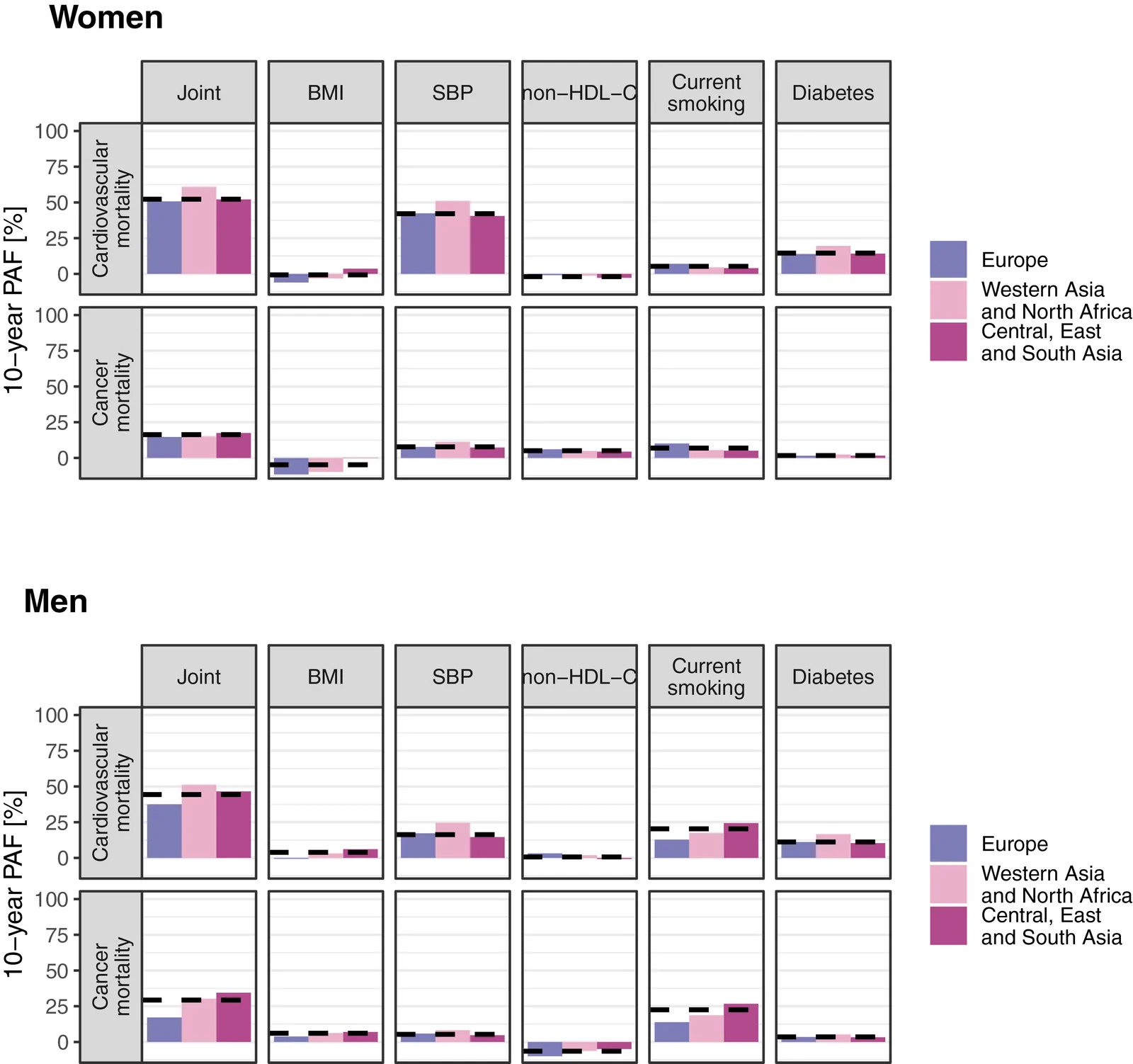
Population-attributable fractions were estimated with the use of Weibull regression models. PAFs for single risk factors and aggregated PAF for the risk factors combined are shown. Abbreviations: SBP, systolic blood pressure; non-HDL-C, non-high-density lipoprotein cholesterol; BMI, body-mass index; MENA, Middle East and North Africa.

For BMI, SBP, and non-HDL-C, age-specific hazard ratios were very similar in women and men. In contrast, associations for current smoking and diabetes differed by sex and outcome. Relative risks for cardiovascular mortality were higher at younger ages in both sexes and attenuated with age, whereas associations with cancer mortality were generally more stable across the lifespan (Supplementary Fig. S6). Hazard ratios from separate exposure–outcome Cox regression analyses, adjusted for age and predefined exposure-specific confounders, were largely consistent with those obtained from the primary multivariable models (Supplementary Table S8). Effect estimates were generally slightly stronger, particularly for diabetes and current smoking; however, the overall pattern of associations remained unchanged.

Linear Cox models suggested regional heterogeneity, particularly among Central, East and South Asian men (Supplementary Fig. S7). Nonlinear models showed regional differences in Western Asia and North Africa and Central, East and South Asia, primarily for cancer mortality in both sexes (Supplementary Fig. S8). Hazard ratios from linear Cox models for non-cardiovascular and non-cancer mortality showed larger associations for diabetes for both women and men for non-cancer mortality (Supplementary Fig. S9) compared to associations with cardiovascular and cancer mortality (Supplementary Fig. S7).

Results were consistent across sensitivity analyses excluding UK biobank, additionally adjusting for birth cohorts and evaluating potential temporal heterogeneity (Supplementary Figs. S9–S12). Unadjusted PAFs showed some differences in estimations for diabetes and BMI for both outcomes and sexes (Supplementary Fig. S13).

### Population-attributable fractions for cardiovascular and cancer mortality

Across sexes and regions, aggregate PAFs were higher for cardiovascular than for cancer mortality (Fig. 2, and Supplementary Table S9). Among women, the five risk factors jointly accounted for a substantially larger proportion of cardiovascular than cancer deaths. Among men, this difference was smaller. Systolic blood pressure and diabetes contributed most to attributable cardiovascular mortality across regions, whereas current smoking was the leading contributor to cancer mortality, particularly among men. Supplementary Fig. S14 shows regional PAF estimates alongside regional current health expenditure per capita.

**Fig. 2:**
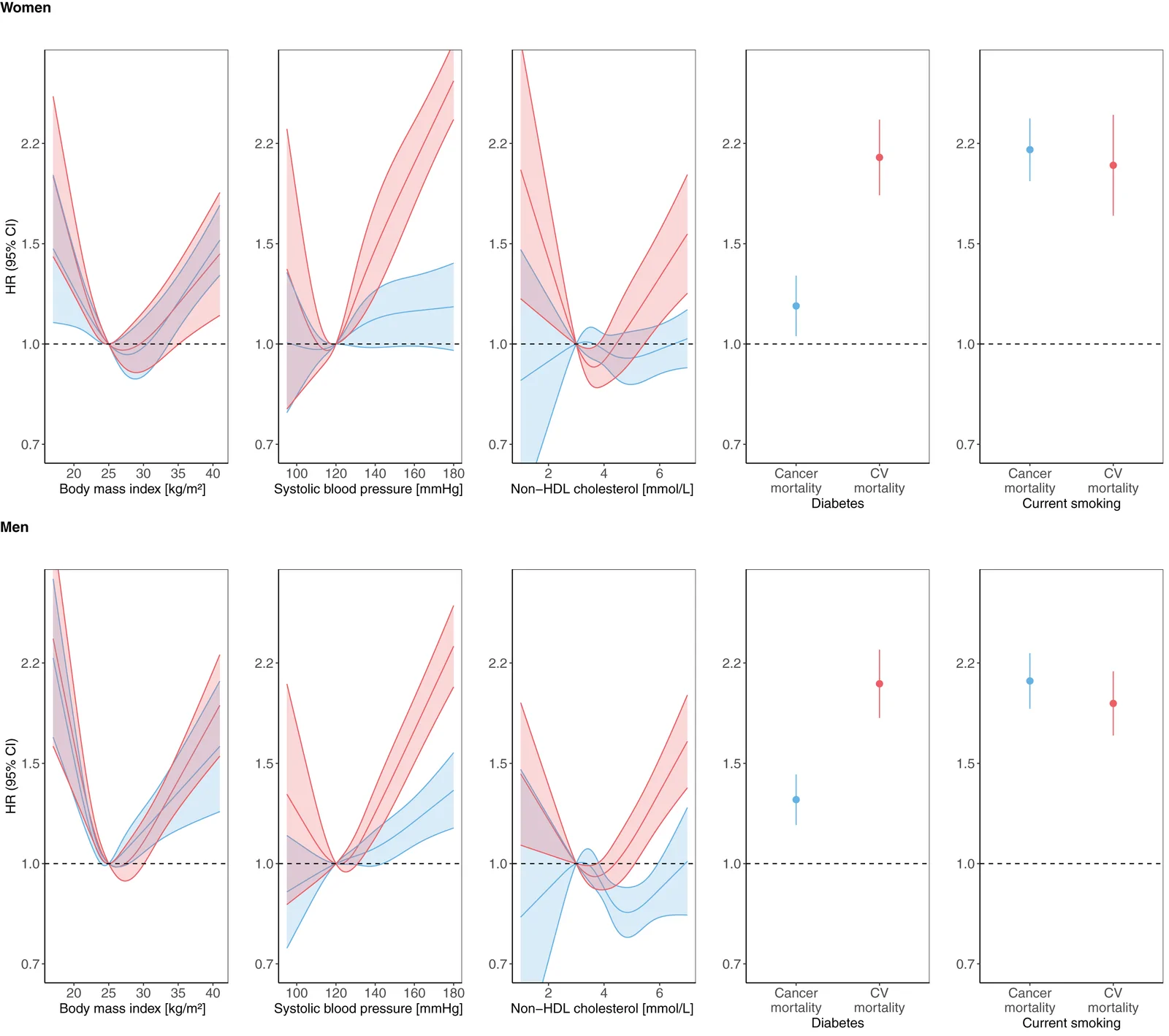
Shown are the results of a 1-year landmark analysis allowing for nonlinear effects. Age was used as the time scale. All five risk factors, together with the use of antihypertensive medications, were included as covariates in the Cox proportional hazards models. Continuous risk factors were modelled using cubic splines. The widths of the 95% confidence intervals are displayed as shaded areas. The p-value shown is for a test for the equality of the covariate's coefficients across the two endpoints. The red line refer to cardiovascular mortality and the blue ones to cancer mortality. Abbreviation: CV mortality, cardiovascular mortality.

When continuous modifiable risk factors were categorised (Supplementary Table S10), notable heterogeneity in PAF estimates emerged. The most pronounced differences were observed for BMI: underweight individuals had the highest PAFs for both cardiovascular and cancer mortality (women: 4.8%, 95% CI 1.2–8.4 and 3.9%, 95% CI 0.6–7.3, respectively; men: 5.4%, 95% CI −0.4 to 11.2 and 4.8%, 95% CI 0.8–8.8). Elevated non-HDL-C (≥5 mmol/L) showed higher PAFs for cardiovascular mortality (women: 2.0%, 95% CI −1.2 to 5.1; men: 3.0%, 95% CI 0.9–5.1), highlighting that contributions to population risk vary across the spectrum of continuous risk factors.

### Population-attributable fractions including education

In secondary analyses additionally adjusting for years of completed education, associations were largely consistent with the primary analyses (Supplementary Fig. S14). Lower educational attainment was associated with higher cardiovascular and cancer mortality. Compared with the main analysis, PAF estimates shifted, with larger attributable fractions for BMI and non-HDL-C for cardiovascular mortality in women and an inverse PAF for non-HDL-C in men. Overall, the joint PAF increased slightly (Supplementary Fig. S14 and Supplementary Tables S11 and S12).

### Premature mortality

In analyses of premature mortality based on 20-year risks at an index age of 50 years, PAF estimates were slightly lower than in the primary analyses, with only modest regional differences, most apparent in Europe (Supplementary Fig. S15 and Supplementary Tables S13 and S14). Among women, BMI PAFs changed the most and were no longer inverse (1.8%, 95% CI −7.5 to 11.0) for cardiovascular mortality and were less strongly inverse for cancer mortality (−3.2%, 95% CI −10.4 to 4.0). Regionally, the largest changes were seen in European women, with a larger decrease for diabetes and cardiovascular mortality (3.5%, 95% CI −0.1 to 7.1) and less pronounced estimates for SBP and cancer mortality (5.7% 95% CI −1.1 to 12.5). Among men, smoking PAFs increased for both outcomes (23.7%, 95% CI 17.9–29.5 for cardiovascular and 26.6%, 95% CI 20.7–32.4 for cancer mortality), and the inverse relation between non-HDL-C and cancer mortality in men (−6.0%, 95% CI −13.2 to 1.1) was less pronounced. Adding educational attainment did not materially change estimates in men, but among women in Europe it lowered estimates of cardiovascular mortality, mainly for systolic blood pressure (41.1%, 95% CI 26.8–55.4) and diabetes (7.1%, 95% CI 2.5–11.7) (Supplementary Tables S15 and S16).

## Discussion

In this large analysis of 798,867 individuals from 25 cohorts across Europe, Western Asia and North Africa, and Central, East and South Asia, we report four main findings: First, a limited set of modifiable risk factors accounted for a substantially larger proportion of cardiovascular mortality than cancer mortality. Second, cardio-metabolic risk factors, particularly SBP and diabetes, were the dominant drivers for cardiovascular mortality, while current smoking was the leading determinant of cancer mortality, especially among men. Third, mortality risks and PAFs varied markedly by sex and region, reflecting differences in risk factor prevalence and baseline risk. Finally, the associations and PAFs were modified by socioeconomic context like education.

The observed regional and sex differences in risk-factor prevalence, mortality, and PAFs underscore that prevention priorities are context dependent. Across regions, men had higher cardiovascular and cancer mortality than women, but the contributions of individual risk factors differed by outcome, sex, and geography, consistent with differences in exposure histories, socioeconomic conditions, and baseline risk.^18^, ^19^, ^20^

These findings inform interpretation and public health relevance. For cardiovascular mortality, the predominant contributions of cardiometabolic risk factors underscore the central role of blood pressure control,^21^, ^22^ and diabetes prevention and management at the population level. BMI showed heterogeneous, sometimes inverse associations across regions; similar patterns have been described previously and may reflect differences in body composition, reverse causation from illness-related weight loss, and mediation through blood pressure and diabetes when these intermediates are modelled simultaneously.^3^, ^23^, ^24^ Notably, categorization of continuous risk factors revealed substantial variation within certain subgroups (e.g., underweight individuals or those with very high non-HDL-C) contributing disproportionately to population-attributable mortality. This underscores that summary estimates may mask important distributional effects. Non-HDL-C showed the expected association with cardiovascular mortality, while weaker or inverse^25^ associations with cancer mortality in some strata may reflect treatment effects, competing risks, and residual confounding, especially at older ages.^3^, ^22^ Tobacco use remained the key shared determinant, with particular relevance for cancer mortality, especially among men, consistent with historical patterns of tobacco exposure.^5^

The PAF estimates highlight clear public health priorities. Scalable strategies focused on detection and treatment of elevated blood pressure and on diabetes control are likely to yield the largest reductions in cardiovascular mortality.^26^, ^27^ For cancer mortality, the smaller aggregate attributable fractions for the examined risk factors are consistent with the multifactorial etiology of cancer and the contribution of additional determinants not captured here, including occupational and environmental exposures, infections, and access to screening and treatment.^5^, ^28^ Together, these results support a dual approach: targeted cardiometabolic risk reduction to lower cardiovascular mortality, alongside comprehensive tobacco control and broader multisectoral cancer prevention strategies.

Equity and generalizability warrant emphasis. European cohorts contributed the largest share of participants, and findings for Western Asia and North Africa, and Central, East, and South Asia should be interpreted in the context of within-region heterogeneity and underrepresentation of some populations and countries. In addition, population-based cohorts tend to represent healthier individuals with lower mortality than the general population of their respective countries, reflecting differences in the prevalence of risk factors, including those examined in this analysis, as well as differences in access to healthcare.^29^ Differences in health-system capacity likely influence both exposure distributions and the translation of risk into mortality, particularly where sustained access to primary care, essential medicines, and cancer screening is limited. Current health expenditure per capita was included as a contextual indicator of health-system resources, and PAF estimates should therefore be interpreted in the context of the capacity to effectively detect, treat, and control modifiable risk factors. These considerations strengthen the rationale for prioritization of cost-effective, scalable prevention strategies–routine blood pressure measurement, affordable and widely available antihypertensive therapy, diabetes detection and management, and sustained tobacco control–especially in resource-constrained settings.^30^

Although the primary analysis assessed overall mortality, these associations are relevant to premature mortality because the effects of smoking, elevated blood pressure, and diabetes are typically strongest early in the life course and decrease with age.^3^ Risk reduction at younger ages may therefore accelerate progress toward global targets for reducing premature non-communicable disease mortality and support the Sustainable Development Goals. Changes in the estimated impact of BMI and non-HDL-C across strata may reflect residual confounding, reverse causation, or treatment and survivorship patterns; future work should evaluate these mechanisms and explicitly quantify premature mortality.

A key strength of this study is the comparison of cardiovascular and cancer mortality within a single, multi-national harmonized dataset, extending evidence from major international efforts.^3^ With a large sample size, prospective follow-up, harmonized individual-level data and consistent analytic methods across regions and sexes, the results help delineate where integrated prevention is most feasible (tobacco control and cardiometabolic risk reduction) and where additional disease-specific strategies remain essential.

Nevertheless, several limitations should be acknowledged. Despite the large sample size, cohort representation was uneven, with a predominance of European participants, and clustering cohorts into broad geographic regions may have obscured within-region heterogeneity.^31^ At the same time, the prospective cohorts from Western Asia and North Africa represented only a small proportion of countries in that region. Results from Western Asia and North Africa should therefore be interpreted with caution. Cardiovascular risk estimates for Central, East and South Asian populations may differ due to population-specific metabolic and genetic characteristics not captured in conventional risk factor models.^31^ Accordingly, regional differences in mortality estimates should be interpreted as descriptive rather than causal, because they may be influenced by differences in baseline risk factor distributions, demographic structure, and underlying health-system characteristics. Furthermore, risk factors were assessed at baseline only, thereby assuming that exposures remained constant over time. However, key risk factors such as blood pressure, BMI, smoking behaviour, and diabetes status may change during follow-up, leading to time-dependent exposure misclassification. This misclassification is likely to be non-differential and may have introduced regression dilution bias, thereby attenuating the observed associations. Additionally, cohorts were initiated and conducted over several decades, during which substantial changes occurred in cardiovascular and cancer prevention, risk factor prevalence, diagnostic practice, access to care, and availability of novel treatments. This temporal heterogeneity may have influenced the distribution of risk factors at baseline and their associations with mortality across cohorts and regions.

Despite extensive harmonisation using predefined variable definitions, recoding procedures, and a dedicated validation process, some residual heterogeneity across cohorts cannot be excluded. Differences in measurement methods, data collection procedures, and underlying population characteristics might have introduced misclassification or variability that could have influenced the results. In addtion, residual confounding cannot be excluded because some relevant risk factors, such as alcohol consumption, were not considered, and outcome misclassification across cohorts remains possible despite the use of ICD codes. Furthermore, given the interrelated nature of cardiovascular risk factors, we cannot fully exclude some degree of overadjustment in our multivariable models, as some variables may lie on causal pathways between other exposures and outcomes. Consequently, these models should be interpreted as estimating mutually adjusted associations rather than the total causal effects of individual risk factors. However, the exposure-specific analyses using predefined confounder adjustment showed consistent results. Premature mortality analyses were limited to individuals aged 50 years at baseline to maintain comparability within the PAF analyses. Consequently, a selection bias cannot be excluded, and the PAF results for premature mortality may have been underestimated. Finally, current health expenditure per capita was used as a proxy for health system resources. However, this measure does not fully capture regional differences in purchasing power, workforce availability, access to care, or health-system efficiency, which might limit comparability across settings.

### Conclusion

Overall, across Europe, Western Asia and North Africa, and Central, East and South Asia, cancer mortality exceeded cardiovascular mortality in women and men, while modifiable risk factors accounted for a larger proportion of attributable cardiovascular than cancer deaths. Regional and sex differences in the magnitude and distribution of associations support context-specific, equity-oriented prevention strategies. Prioritizing low-cost, high-impact interventions to improve blood pressure control, prevent and manage diabetes, and reduce tobacco use could meaningfully reduce premature mortality and advance progress toward global non-communicable disease prevention goals.

## Contributors

Analyses were performed by FMO. TL and FMO accessed and verified the underlying data. The first draft of the manuscript was prepared by TL, FMO, CM, and SB, and reviewed and edited by all authors. All authors jointly agreed to submit the manuscript for publication and vouch for the accuracy and completeness of the data.

## Data sharing statement

No data sharing is planned due to the restrictions of data transfer agreements with the participating cohorts. Upon reasonable request and with approval of the respective data providers, access to de-identified data may be considered.

## Declaration of interests

GE declares owning shares of AstraZeneca. SG declares participation on a Data Safety Monitoring Board for the AGP3 (“Ambroxol in Type III Gaucher Disease”) study, ClinicalTrials.gov ID NCT07285369, as DSMB Member for Ambroxol in Type III Gaucher Disease (GD3): A Prospective 6-Month Single-Center Open-Label Study with an optional 12-month extension phase, Agyany Pharma LTD, no payments received. DPL declares consulting fees from Beigene, Boston Scientific, and Bayer, and payment or honoraria for lectures, presentations, speakers bureaus, manuscript writing or educational events from AstraZeneca and Novartis. MNL declares having served on the Scientific Advisory Board for Roche Diagnostics. PM declares that his lab is funded by the British Heart Foundation, NextGenerationEU PNRR, Heart Research UK, Italian Ministry of University PRIN, BBSRC International Partnerships Funding, European Commission/Erasmus+, and COST (European Cooperation in Science and Technology), and that he is the Vice-President for the British Pharmacological Society (BPS), Vice-Chair of the Basic and Translational Section for the International Union of Basic and Clinical Pharmacology (IUPHAR), Chair of the Translational Research Medical Review Panel for Heart Research UK (HRUK), a Nucleus Member of the European Society of Cardiology (ESC) Working Group on Atherosclerosis & Vascular Biology, a member of the Executive Committee of the British Atherosclerosis Society (BAS), Chair of the Publication Committee of the International Union of Immunological Societies (IUIS), a member of the Translational Clinical Studies Grant Panel for the Chief Scientist Office (CSO), and co-Director of the ARUA/Guild Africa-Europe CoRE in Non-Communicable Diseases & Multimorbidity. RT declares research support from the German Center for Cardiovascular Research (DZHK), the Kühne Foundation, the Joachim Herz Foundation, the Swiss National Science Foundation (Grant No P300PB_167803) and the Swiss Heart Foundation, payment for expert testimony from Roche Diagnostics, and that he has served on Scientific Advisory Board for Roche (not related to the current work), and honoraria from Abbott, Amgen, AstraZeneca, Psyros, Roche, Siemens, Singulex, SpinChip, and Thermo Scientific BRAHMS, and is holder of a patent for international application on a computing device for estimating the probability of myocardial infarction (WO2022043229A1), and serving on an advisory board for Amgen, and is co-founder and shareholder of the ART-EMIS Hamburg GmbH. APa declares support from Jagiellonian University Medical College (N43/DBS/000272). SB declares a leadership or fiduciary role as President German Society of Cardiology and Board member Global Cardiovascular Research Funder Forum. FMO is listed as co-inventor of an international patent (pending) on the use of a computing device to estimate the probability of myocardial infarction (International Publication Number WO2022043229A1) and is a shareholder of the ART-EMIS Hamburg GmbH. CM reports receiving speaker fees and travel support from companies involved in the development and manufacture of antihypertensive medications (Alnylam), and serving on a Clinical Research Steering Committee for Alnylam, all outside the submitted work. All other authors declare no conflict of interests.
