# Supplementary material for: Effect of modifiable risk factors on cardiovascular and cancer mortality across European and Asian regions: a multicohort study
Source: Lancet Reg Health Eur. 2026 Nov 9;72:101918. doi: 10.1016/j.lanepe.2026.101918 (PMC13760709; doi:10.1016/j.lanepe.2026.101918)
Supplement: Supplementary file 1 [file mmc1.docx]

**Supplementary material**

Supplement to: Lorenz, T, et al. Effect of Modifiable Risk Factors on Cardiovascular and Cancer Mortality across European and Asian regions - A multicohort study

This appendix has been provided by the authors to give readers additional information about the work.

Content

[Collaborators	4](#_Toc233107642)

[Supplementary Methods	4](#_Toc233107643)

[Study population	4](#_Toc233107644)

[Inclusion of cohorts	4](#_Toc233107645)

[Risk factor and outcome definitions	4](#_Toc233107646)

[Data Harmonization processes	4](#_Toc233107647)

[Statistical Methods	4](#_Toc233107648)

[Description of longitudinal population-based cohorts	1](#_Toc233107649)

[Description of health examination survey data	9](#_Toc233107650)

[Supplementary results	15](#_Toc233107651)

[Supplementary figures	15](#_Toc233107652)

[Figure S1: Study flow chart	16](#_Toc233107653)

[Figure S2: Analytical workflow	17](#_Toc233107654)

[Figure 3a: Cumulative incidences for cardiovascular mortality (red lines) and cancer mortality (blue lines) using age as time scale by sex.	18](#_Toc233107655)

[Figure S3b: Cumulative incidences for cardiovascular mortality (red lines) and cancer mortality (blue lines) using time since study entry as scale by sex	19](#_Toc233107656)

[Figure S4: Cumulative incidences for cardiovascular mortality (red lines) and cancer mortality (blue lines) using age as time scale a) with UK Biobank and b) without UK Biobank by sex.	20](#_Toc233107657)

[Figure S5: Associations of risk factors with cardiovascular (red lines) and cancer mortality (blue lines) allowing for effects to change with age by sex.	21](#_Toc233107658)

[Figure S6: Risk factor associations for cardiovascular and cancer mortality from linear Cox models by region and sex.	22](#_Toc233107659)

[Figure S7: Risk factor associations with cardiovascular disease (red) and cancer mortality (blue) including education by region and sex.	23](#_Toc233107660)

[Figure S8: Risk factor associations for non-cardiovascular and non-cancer mortality from linear Cox models by sex and region.	24](#_Toc233107661)

[Figure S9: Risk factor associations with cardiovascular disease (red) and cancer mortality (blue) adjusting for examination year, by sex and year.	25](#_Toc233107662)

[Figure S10: Risk factor associations for cardiovascular and cancer mortality from linear Cox models by sex and region, adjusting for examination year.	26](#_Toc233107663)

[Figure S11: Risk factor associations for cardiovascular and cancer mortality from linear Cox models, by region, in cohorts recruited after 1988.	27](#_Toc233107664)

[Figure S12: Population-attributable fractions for cardiovascular and cancer mortality, excluding the UK Biobank by region and sex	28](#_Toc233107665)

[Figure S13: Unadjusted population-attributable fractions for cardiovascular and cancer mortality	29](#_Toc233107666)

[Figure S14: A) Joint population-attributable fractions of five risk factors for 10-year incidence of cardiovascular and cancer mortality by sex and region, and B) Current healthcare expenditure per capita and region.	30](#_Toc233107667)

[Figure S15: Population-attributable fractions of modifiable risk factors and education for cardiovascular and cancer mortality by geographic region and sex	31](#_Toc233107668)

[Figure S16: Population-attributable fractions of modifiable risk factors for premature cardiovascular and cancer mortality by geographic region and sex	32](#_Toc233107669)

[Supplementary tables	33](#_Toc233107670)

[Table S1: STROBE statement for cohort studies - completed checklist.	33](#_Toc233107671)

[Table S2: Core variables of interest	35](#_Toc233107672)

[Table S3: End point definitions	35](#_Toc233107673)

[Table S4: Variable Definitions and Coding	35](#_Toc233107674)

[Table S6: Health examination surveys' baseline characteristics according to geographic region	40](#_Toc233107675)

[Table S7: Cohort studies' follow-up information on cardiovascular and cancer mortality	41](#_Toc233107676)

[Table S8. Risk factor-specific associations with cardiovascular and cancer mortality using individual Cox regression models by sex.	42](#_Toc233107677)

[Table S9: Population-attributable fractions of modifiable risk factors for cardiovascular and cancer mortality by geographic region and sex	43](#_Toc233107678)

[Table S10: Population-attributable fractions for cardiovascular and cancer mortality across categories of continuous modifiable risk factors	44](#_Toc233107679)

[Table S11: Population-attributable fractions of modifiable risk factors and education for cardiovascular and cancer mortality by geographic region and sex	45](#_Toc233107680)

[Table S12: Population-attributable fractions across categories of continuous modifiable risk factors and education for cardiovascular and cancer mortality	46](#_Toc233107681)

[Table S13: Population-attributable fractions for premature cardiovascular and cancer mortality by geographic region and sex	47](#_Toc233107682)

[Table S14: Population-attributable fractions across categories of continuous classic modifiable risk factors for premature cardiovascular and cancer mortality	48](#_Toc233107683)

[Table S15: Population-attributable fractions of classic modifiable risk factors and education for premature cardiovascular and cancer mortality by geographic region and sex	49](#_Toc233107684)

[Table S16: Population-attributable fractions across categories of continuous modifiable risk factors and education for premature cardiovascular and cancer mortality	50](#_Toc233107685)

[References	52](#_Toc233107686)

# Collaborators

(In alphabetical order)

**Mahshid Ghasempour Moghaddam** (University Medical Center HamburgEppendorf, Hamburg, Germany)**, Elisa Großmann** (University Heart & Vascular Center Hamburg, Hamburg, Germany; Population Health Research Department, University Heart & Vascular Center Hamburg, University Medical Center HamburgEppendorf, Hamburg, Germany), **Linlin Guo** (University Heart & Vascular Center Hamburg, Hamburg, Germany; Population Health Research Department, University Heart & Vascular Center Hamburg, University Medical Center HamburgEppendorf, Hamburg, Germany), **Davood Khalili** (Prevention of Metabolic Disorders Research Center, Research Institute for Metabolic and Obesity Disorders, Research Institute for Endocrine Sciences, Shahid Beheshti University of Medical Sciences, Tehran, Iran), **Marie Neumann** (University Medical Center HamburgEppendorf, Hamburg, Germany), **Tetsu Watanabe** (Department of Cardiology, Pulmonology, and Nephrology, Yamagata University School of Medicine, Yamagata, Japan)

# Supplementary Methods

## Study population

## Inclusion of cohorts

The cohorts included in this study were identified through a literature review. We evaluated all relevant publications up to June 2022 using PubMed, Web of Science, and Google as search engines. The search strategy included terms such as population-based, longitudinal, cardi* disease, cerebr* disease, stroke, coronary, heart disease, myocardial infarction, as well as cancer, neoplas*, and oncolog*, in addition to biosamples, biomarkers, lipids, cholesterol, mortality, and incidence. Existing collaborations among study investigators and the availability of variables of interest (**Table S2**) were also used to identify cohorts.

## Risk factor and outcome definitions

An overview of all variables of interest are provided in **Table S2**. Systolic blood pressure at baseline was based on each cohort’s study protocol for. Non-high-density lipoprotein cholesterol (non-HDL-C) was calculated as total cholesterol minus HDL cholesterol, based on blood samples taken at baseline following the cohort-specific laboratory procedures. Body mass index was based on each cohort’s study protocol. where available, it was calculated using measured height and weight obtained at baseline.

Current smoking was defined as regular (≥1 cigarette per day) or occasional (<1 cigarette per day) use of cigarettes, cigars, cigarillos, or pipe, but excluded chewing tobacco. Depending on each cohort’s assessment, diabetes was defined based on medical records, self-reported participant questionnaires, or newly diagnosed at baseline. Where applicable diabetes was defined by cohort-specific measurements of hyperglycemia. Education was defined as completed years of education, and cohort-specific tertiles were calculated. When education was reported as degrees only, these were converted to the equivalent number of years. The definitions of study endpoints are provided in **Table S3**.

## Data Harmonization processes

For this project, we defined a set of core variables of interest (**Table S2 and Table S3**), on the basis of Multinational Monitoring of Trends and Determinants in Cardiovascular Diseases (MONICA)(1)/ MONICA Risk Genetics Archiving and Monograph(MORGAM)(2) standards (**Table** **S4**). These definitions were applied uniformly across cohorts whenever possible. Participant age was recorded at the time of examination in years, and sex was coded as male or female. All datasets were transferred individually, reviewed, and subsequently harmonized in Hamburg based on MONICA/MORGAM definitions. Harmonisation included standardisation of units and coding schemes across cohorts, together with predefined harmonisation rules. Each dataset was harmonized in R using a scripted pipeline to ensure reproducibility and was independently validated by a second data analyst. Automated quality-control procedures were implemented, including range and plausibility checks, detection of logical inconsistencies, and cross-cohort distribution assessments. Visual inspection of key variables was additionally performed to ensure consistency across datasets. This process is further depicted in Figure S2.

## Statistical Methods

Multiple imputation by chained equations(3) was performed separately for each study and for each sex. Follow-up data for the end points considered were included in the imputation models using the event indicator and the Nelson-Aalen estimate of the cumulative hazard function, as recommended by White et al.(4). Ten imputed datasets were generated for the analysis.

Continuous variables are reported as quartiles or minimum and maximum, while binary variables are presented as absolute and relative frequencies. Regional baseline characteristics were estimated with (Table S4) direct standardization according to the age and sex distribution of the entire dataset. The following age groups were used in the standardization: age ≤40 years; >40 to ≤45; >45 to ≤50; >50 to ≤55; >55 to ≤60; >60 to ≤65; >65 to ≤70; and >70 years.

The median (25^th^, 75^th^ percentile) duration of follow-up was computed using the Kaplan-Meier potential follow-up estimator.(5)

For each sex, ten-year incidences and events per 1000 person years were estimated. Direct standardization based on the age distribution of the entire dataset for each sex, was used when computing these estimates for each geographic region. Poisson regression models with follow-up time that was log-transformed as an offset were used in the estimation of the events per 1000 person years.

For all the models, coefficients were pooled across studies by region, as well as over all datasets, using a multivariate random effects meta-analysis. Specifically, sex-specific Cox models were computed for each study. All models used age as the time scale.(6) In addition to age, the following variables were included in all models: body-mass index (BMI), systolic blood pressure (SBP), non-high-density lipoprotein (HDL) cholesterol, current smoking, diabetes, use of antihypertensive medications, and for the secondary analysis years of completed education. The use of lipid-lowering medications was not included in the models because data was missing for approximately 13% of individuals overall and was completely missing in some cohorts. Different sets of models were computed: 1) All covariates were entered into the model linearly. 2) Continuous covariates (BMI, SBP, and non-HDL cholesterol) were modeled using restricted cubic splines with four knots, with the same knots applied across all studies. 3) The last set of models allowed for the effects to change with age (the time scale). In the event that hazard ratios do not remain constant over age, the hazard ratio can be interpreted as an age-averaged effect.(7) A one-year landmark analysis was applied in all models, including only individuals surviving at least one year in the computations. To compare two different time-to-event endpoints, which are regressed on the same set of covariates, in our case, cardiovascular and cancer mortality, we created a stacked dataset by combining two copies of the original data.(8) The predictor information remains the same in both copies, but the time-to-event endpoint differs. Additionally, an indicator variable is added to distinguish between the two datasets (or, equivalently, the endpoints).

Population-attributable fractions (PAF) for the 10-year incidence of cardiovascular and cancer mortality were estimated using the methods from Laaksonen and colleagues.(9) This approach allows the estimation of population-attributable fractions for combinations of risk factors. For the approximation of premature death, 20-year incidence of cardiovascular and cancer mortality at baseline age 50 were computed. Time-to-event models, similar to those used in the association analyses but with categorized covariates, were developed using the cohort studies and then applied to each individual in the contemporary health examination surveys to estimate the population-attributable fractions.

PAF estimates the proportion of the outcomes that could be prevented if some of the risk factors could be changed to given target values. The computations were based on the following formula(9):

$$PAF\left( t \right)=1-\frac{\sum_{i=1}^{n} P\left\{ T_{i}^{event}\leq min\left( T_{i}^{M},t \right) | X_{i}^{*} \right\}}{\sum_{i=1}^{n} P\left\{ T_{i}^{event}\leq min\left( T_{i}^{M},t \right) | X_{i} \right\}},$$

where $T_{i}^{event}$is the time to event for individual $i$; $T_{i}^{M}$is the time to death for individual i; $X_{i}$ are the covariate values for individual $i$; $X_{i}^{*}$ are the covariate target values for individual $i$; $P\left\{ T_{i}^{event}\leq min\left( T_{i}^{M},t \right) | X_{i} \right\}$ is the probability of developing event before time $t$ given the covariates $X_{i}$ accounting for death as competing event; $P\left\{ T_{i}^{event}\leq min\left( T_{i}^{M},t \right) | X_{i}^{*} \right\}$ is the probability of developing event before time $t$ given the target covariates $X_{i}^{*}$ accounting for death as competing event. This approach gives the estimation of PAF for combinations of risk factors, without producing estimates that exceed 100%.

Weibull proportional hazard models were used to estimate the 10-year event probabilities required for the PAF estimation. Specifically, cause-specific Weibull models were applied, treating death before cardiovascular or cancer mortality as competing events. As it was done with the Cox regressions, a two-stage random effects multivariate individual participant data meta-analysis was conducted. Age was used as the time scale, and the following variables were included simultaneously in the models: BMI (categorized), SBP (categorized), non-HDL cholesterol (categorized), current smoking, diabetes, and use of antihypertensive medications. BMI was grouped according to an adapted version of the WHO classification (BMI <20 kg/m^2^; 20 to <25 kg/m^2^ (reference category); 25 to <30 kg/m^2^; ≥30 kg/m^2^). The cut-off value of 20 instead of 18.5 kg/m^2^ was used because of the low number of individuals with BMI <18.5kg/m^2^, as was done by Leong et al.(10) SBP was categorized as <120 mmHg (reference category), 120 to <140 mmHg, 140 to <160 mmHg, and ≥160 mmHg; and non-HDL cholesterol as < 116.01 mg/dL (reference category), 116.01 to < 154.68 mg/dL , 154.68 to < 193.35 mg/dL, and ≥ 193.35 mg/dL. Current smoking and diabetes were used as binary variables (yes/no), with the “no” category as the reference. For the secondary analyses, years of completed education were divided into tertiles, with the highest tertile as reference. The distributional assumptions of the Weibull model were checked for each endpoint, cohort, and sex, as described in Harrell.(11) Specifically, the estimated survival function of the standardized residuals was plotted alongside the survival function of the assumed distribution, with the Kaplan-Meier estimator used for the former. The models did not allow for time-varying coefficients, and we are not aware of any implementation that allows for the computation of probabilities for such models. As the population-attributable fractions vary with the prevalence of the risk factor, which may have changed over time, as may have risk factor management,(12,13) we used contemporary cross-sectional national health examination surveys (from 2010 onward) to estimate contemporary risk factor prevalence when calculating the population-attributable fractions. **Figure S1** provides an overview of the included cohorts, countries and participants. Each national health examination survey is described above. We apply the term “cohort study” to refer to those population-based cohort studies employed in time-to-event analyses and the term “health examination survey” to refer to the surveys and studies of a cross-sectional nature used for contemporary risk factor prevalence. The pooled Weibull models were applied to the individuals in the health examination surveys. Specifically, the parameters of the models used to compute the 10-year probability of event were estimated using the cohort studies data, but the actual probabilities used in the formula above were estimated in the health examination surveys. For this purpose, only the global models were used. Confidence intervals were computed with help of the bootstrap, with 250 repetitions, as follows: first the parameters of the pooled Weibull models were sampled from a multivariate normal distribution using the mean vector and covariance matrix estimated in the original data; afterwards the new models obtained this way were applied to a dataset drawn with replacement from the health examination surveys; and then PAFs were computed based on the bootstrapped Weibull parameters and bootstrapped health examination surveys. The methods used allow for the estimation of PAFs for single risk factors or any combination of them, including an aggregated PAF in which all risk factors are set to their target values simultaneously. A one-year landmark analysis was applied in the computation of the Weibull models.

For all time-to-event analyses, individuals with cardiovascular disease or cancer at baseline were excluded.

## Description of longitudinal population-based cohorts

**Europe**

| Country | Study | Study/cohort full name and short description |
| --- | --- | --- |
| Czechia, Poland | HAPIEE CZ, HAPIEE PL | Health, Alcohol and Psychosocial factors in Eastern Europe (HAPIEE): The HAPIEE study comprises four prospective urban population-based cohorts from Eastern Europe, including Novosibirsk (Russia), Krakow (Poland), Kaunas (Lithuania), and two cities of the Czech Republic. Each cohort recruited a random sample of men and women age 45-69 years at baseline in May 2002 (August 2006 in Lithuania), stratified by sex and 5-year age group. Participants were selected from population registers (electoral roll list in Russia). Participants completed extensive questionnaires, underwent examination in a clinic and provided a blood sample. The sample size for HAPPIE CZ is 8,857 (response rate 55%) and for HAPIEE PL is 10727 (response rate 61%). Deaths in the cohorts were identified by linkages with national or regional death registers. Follow-up is completed to 2024 in Czechia and to 2021 in Poland. All participants gave written informed consent.  For this Analyses only HAPIEE CZ and PL were used.  The HAPIEE study was funded by the Welcome Trust (grant WT064947 and WT081081), the US National Institute of Aging (grant R01 AG23522), and the MacArthur Foundation.  Data from the HAPIEE CZ cohort were provided by the study team under the leadership of Martin Bobak (PI). The Czech component of HAPIEE was supported by the Programme JAC project SALVAGE [CZ.02.01.01/00/22_008/0004644], funded by the Ministry of Education, Youth and Sports of the Czech Republic and co-funded by EU; by EU’s H2020 research and innovation program (grant 857560, CETOCOEN Excellence); by AZV (grant NW26-02-00294); and by RECETOX Research Infrastructure (LM2023069). The European Commission is not responsible for the content.  The Polish component was supported by the grant of Polish National Science Centre (2018/29/B/NZ7/02118) and Jagiellonian University Structural Grant (N42/DBS/000272).  <http://www.ucl.ac.uk/easteurope/hapiee.html> |
| Denmark | CCHS | The Copenhagen City Heart Study is a large prospective cardiovascular population study of 19,000 women and men between 20–93 years old, that was launched in 1975. The source population consisted of about 90,000 adults (≥20 years old) living in a defined area in Copenhagen, Denmark. These were identified through the Copenhagen Population Register using a national registration number. The original purpose of the study was to focus on prevention of coronary heart disease and stroke. During the years many other aspects, such as pulmonary diseases, heart failure or arrhythmia, have been added to the study. CCHS conducted 4 stages of follow-ups, namely, from 1976 to 1978, 1981-1983, 1991-1994 and 2001-2003. End points were ascertained through record linkage with the National Patient Register, the Civil Registration System, and the Cause of Death Registration. All participants gave written informed consent.  <https://clinicaltrials.gov/ct2/show/NCT02993172> |
| Germany | ESTHER | The ESTHER study (Epidemiologische Studie zu Chancen der Verhütung, Früherkennung und optimierten Therapie chronischer Erkrankungen in der älteren Bevölkerung [German]) is a prospective population-based cohort study in the federal state of Saarland, Germany. In total, 9,949 men and women (age 50-74 years at baseline) were recruited during a routine health check-up between 2000 and 2002. Fatal events were obtained through linkage with the general population registry and cause of death information was collected from death certificates and physicians who issued the certificate. Non-fatal data were collected via questionnaires that were validated by the participant’s primary care physician. Follow-up was completed by 31st of December 2010. Approval for the study was granted by the Ethics Committees of the Medical Faculty Heidelberg at the University of Heidelberg and the Physicans’ Board of Saarland. All participants provided written informed consent.  <https://www.thl.fi/publications/morgam/cohorts/full/germany/ger-esra.htm> |
| Germany | SHIP | SHIP (Study of Health in Pomerania) is a population-based project conducted in Northeast Germany. The study aims to assess prevalence and incidence of common risk factors, subclinical disorders and clinical diseases and to investigate associations and interactions among them using comprehensive medical assessments. The first SHIP cohort was recruited between 1997 and 2001 and included 4,308 individuals at baseline (SHIP-0, 20-70 years, response 68.8%), 3,300 after five years (SHIP-1) and 2,333 after 11 years (SHIP-2). In parallel to SHIP-2, baseline examinations of a second, independent cohort (SHIP-TREND) were conducted in 4,420 participants (20- 79 years, response 50.3%). For the current analysis only data of SHIP-0 was used. Data were transferred from Greifswald. The harmonization was implemented in Hamburg. SHIP is one of the MORGAM/BiomarCaRE cohort studies and one of the population-based projects with very comprehensive examinations including interviews, cardio-metabolic ultrasound exams, cardiopulmonary exercise tests and whole-body magnetic resonance imaging in a general population setting. In addition to the examination follow-ups, information on fatal and non-fatal disease is collected on a regular basis. However, information of incident CVD is lacking. Mortality follow-ups are conducted semi-annually by record linkage with data bases of the regional population registry. Causes of death are defined from the official death documents provided by regional health authorities. Active follow-ups for non-fatal diseases are performed biannually and by interviews during follow-up examinations every five years. All participants provided written informed consent.  <https://www.thl.fi/publications/morgam/cohorts/full/germany/ger-grea.htm>  <https://academic.oup.com/ije/article/51/6/e372/6555287> |
| Italy | Moli-sani | The cohort of the Moli-sani study was recruited in the Molise region from city hall registries by multistage sampling. First, townships were sampled in major areas by cluster sampling; then, within each township, participants age 35 years or older were selected by simple random sampling. Exclusion criteria were pregnancy at the time of recruitment, inability to comprehend, current multiple trauma or coma, or refusal to sign the informed consent. A total of 24,325 men (48%) and women (52%) were examined at baseline from 2005 to 2010. Participation was 70%. Informed consent was obtained from all participants.  The cohort was followed up for a median of 13.1 years (maximum 15.8 years) by December 2020. Follow-up is achieved through record linkage to regional mortality register and hospital discharge registers; validation of events was achieved through hospital record linkage and doctor’s medical records using updated MORGAM criteria.  https://www.moli-sani.org  <https://www.thl.fi/publications/morgam/cohorts/full/italy/ita-mola.htm> |
| France | PRIME Lille | The PRIME (Prospective Epidemiological Study of Myocardial Infarction) study examined the classic and putative cardiovascular risk factors to explain the large difference in heart disease incidence between Ireland and France. The study includes four cohorts of men age 50-59; from Belfast, Northern Ireland (N=2,745) and Lille (N=2,633), Toulouse (N=2,610) and Strasbourg (N=2,612) in France. Baseline examinations took place in 1990-1993 and targeted cohorts which had broadly similar social class structures to the background population, initially sampling from industries and various employment groups, employment groups with more than 10% of their workforce of foreign origin were excluded. Follow-up for 10 years for each participant (Toulouse, Strasbourg and Lille) and for 18 years (Belfast) was achieved through annual follow-up questionnaires with verification against national death registers, medical records, hospital discharge diagnoses. Endpoints were systematically validated through medical record review from hospitals and general practitioners, and subsequently adjudicated by an expert medical committee. End points were validated by expert medical committee. Informed consent was obtained from all enrolled participants. For this project, only the PRIME Lille data was used.  <https://www.thl.fi/publications/morgam/cohorts/full/uk/unk-bela.htm> |
| Kyrgyz Republic | Interepid | The international epidemiological project “Interepid” includes cross-sectional data on the prevalence of major non-communicable diseases and risk factors, and a prospective stage among rural residents of two countries — Russia and Kyrgyz Republic. The study was carried out by one protocol with specifically developed surveys in 2011-2012, with a representative selection of rural inhabitants of the Volzhskiy region of Samarskaya Oblast of Russia (n=1050), and of Chuyskaya Oblast of Kyrgyz Republic (n=1341),that included participants age 20-64 years. In 2019, the vital status of respondents was reassessed (7 years after the initial examination), and end points were collected, including all-cause and cause-specific mortality based on medical death certificates.  All participants provided written informed consent.  <https://pubmed.ncbi.nlm.nih.gov/32870173/>  <https://russjcardiol.elpub.ru/jour/article/view/4999?locale=en_US> |
| Norway | HUNT | The Trøndelag Health Study (Helseundersøkelsen i Trøndelag [Norwegian]: HUNT) was initiated in the 1980s as a health survey that addressed four main topics: hypertension, diabetes, quality of life, and tuberculosis as well as other lung diseases. Today, the HUNT study includes more than 229,000 adult Norwegian participants from four surveys (HUNT 1 1984-86; HUNT 2 1995-97; HUNT 3 2006-08, and HUNT 4 2017-2019). Every citizen of Nord-Trøndelag County in Norway aged 20 years or older, has been invited to all the surveys for adults. Participants may be linked in families and followed up longitudinally between the surveys and in several national health- and other registers covering the total Norwegian population. In 2018, the two counties Sør-Trøndelag and Nord-Trøndelag were merged into one county: Trøndelag, and from the fall of 2019, HUNT was expanded to include the entire county. Cause-specific mortality endpoints are followed up through linkage with national registries, including the Norwegian Cause of Death Registry since 2012, which systematically collects mortality data based on medical death certificates. The HUNT Study includes data from questionnaires, interviews, clinical measurements and biological samples (blood and urine). The questionnaires included questions on socioeconomic conditions, health related behaviors, symptoms, illnesses and diseases. For the current analyses, data from 65,242 participants from HUNT 2 was included. All participants provided informed consent.  <https://www.ntnu.edu/hunt> |
| Sweden | Malmö MDCS | The Malmö Diet and Cancer Study (MDCS) was original initiated to clarify whether a western diet is associated with certain forms of cancer whilst taking other life-style factors into account. Individuals age 45-69 years living in Malmö city, Sweden, were eligible to participate. From 1991-1994, every other participant was invited to also take part in a substudy of the epidemiology of carotid artery disease. A total of 6,103 subjects accepted the invitation and where rescheduled for fasting blood sampling under standardized conditions. Risk factors were estimated on the basis of laboratory tests, baseline examinations, and through the questionnaire administered at the baseline visit. Information regarding smoking habits, physical activity, medical history, and use of medications was based on the self‐administered questionnaire. Cause-specific mortality outcomes are available through a record linkage with the Swedish Cause of Death Registry. The study was approved by the Ethics Committee at Lund University. Each participant gave his or her written informed consent.  <https://www.malmo-kohorter.lu.se/malmo-cohorts> |
| The Nether-lands | PREVEND | The PREVEND (Prevention of REnal and Vascular ENd stage Disease) study is a general population-based prospective cohort study that was started in 1997 in the city of Groningen, The Netherlands. For this study all the inhabitants of the city of Groningen were asked, in 1997, to complete a brief questionnaire and provide morning urine. Of the 41,000 respondents, 8,600 persons were immediately invited for further examination. The study is still ongoing, but follow-up on CV mortality has been collected and until 31st December 2016, whereas follow-up on non-fatal CV events has been collected until 31th December 2010. Information on cardiovascular morbidity was collected through linkage with the Dutch national registry of hospital discharge diagnosis (PRISMANT). Mortality data was retrieved from the municipal register and primary cause of death was provided by Dutch Central Bureau of Statistics data based on death certificate information. The PREVEND study was approved by the Medical Ethics Committee of the University of Groningen (approval number: MEC96/01/022). Informed written consent was obtained from all participants. The study adhered to the ethical principles set by the Declaration of Helsinki.  The PREVEND study was supported by The Dutch Kidney Foundation which supported the infrastructure of the PREVEND program from 1997 to 2003 (Grant E.033). The University Medical Center Groningen supported the infrastructure from 2003 to 2006. The Netherlands Heart Foundation provided support for studies on HDL metabolism (Grant 2001.005)  <https://umcgresearch.org/w/prevend> |
| United Kingdom | UK Biobank | The UK biobank is a large-scale prospective study with 502,489 (46%male) participants between 37 and 73 years old, which were recruited in 22 research assessment centers across the UK from 2006 till 2010. The response rate was 5.5%. The UK Biobank aims to create a better understanding, prevention and treatment of a range of different diseases. Besides standardized questionnaires and physical exams, the participants also give biosamples and genetic data. End points are mainly collected through linkage with national datasets, including the death and cancer registry, primary care data and hospital in- and outpatient episodes. The current dataset is followed up through 2018. All participants gave informed consent.  For this project, the data from proposal ID 63412 were used.  This work uses data provided by patients and collected by the NHS as part of their care and support.  <https://www.ukbiobank.ac.uk/> |

**The Western Asia and North Africa**

| Iran | GCS | The Golestan Cohort Study (GCS) is a long-term population-based prospective study in Iran. The primary purpose was to study risk factors of esophageal cancer in a high-risk area in the north-eastern Iran. This population-based cohort enrolled 50045 men and women aged 40-75 years, without history of upper gastrointestinal cancers, from Gonbad City (n=10032) and 326 villages (n=40013) in Golestan Province, between 2004 and 2008. The design and objectives of the GCS have been described previously. In the main phase of the study (enrollment phase), biomarkers were not measured. In the first repeated measurement phase (2010-2012), a random sample of the participants (n=11400; 80% rural and 20% urban participants) were assessed again. Smoking, alcohol use, blood pressure, BMI, drug history, and history of chronic diseases were re-evaluated. In addition, fasting blood sugar, triglycerides, total cholesterol, low-density lipoprotein cholesterol, high-density lipoprotein cholesterol, and creatinine were measured. All participants are being followed up actively every 12 months.  In case of a reported death, the follow-up team collected all hospital documents, such as physician notes, electrocardiography reports, laboratory test results, and radiologic documents from all medical centers in Golestan Province and the neighboring provinces. Causes of death were determined based on ICD-10 codes.  The study protocol and the written informed consent used for this study were approved by the ethical review committees of Digestive Disease Research Centre, Tehran University of Medical Sciences (DDRC), International Agency for Research on Cancer (IARC), and National Cancer Institute (NCI). In June 2008, the accrual goal of 50 000 subjects was reached and enrolment was closed.  GCS was funded by Tehran University/Medical Sciences (81/15 to R.M., Principal Investigator); Cambridge University (Cancer Research UK, C20/A5860) the intramural research program of the NCI, National Institutes of Health; and the IARC.  <https://www.ncbi.nlm.nih.gov/pmc/articles/PMC3709199/> |
| --- | --- | --- |
| Iran | PCS | The Pars Cohort Study (PCS) is a 10-year cohort study aiming to investigate the burden and the major risk factors on non-communicable diseases, and to establish a setting to launch interventions for prevention of these diseases and controlling their risk factors. The participants were recruited between 2012 and 2014 and are from the district of Valashahr. The 9264 participants included, aged 40-75 years, represent both healthy and disease populations of the south of Iran. All participants were actively followed-up for 12 months and then contacted annually for long-term follow-up for at least 10 years. . For cause of death confirmation, two separate internists independently review the existing documents and allocate an ICD-10 code and date of occurrence or death to each participant. If the two codes differ, a third more senior internist reviews the documents and makes a final diagnosis. PCS was developed by the joint collaboration of research teams from Non-Communicable Disease Research Center (NCDRC) in Shiraz University of Medical Sciences (SUMS) and Digestive Diseases Research Institute (DDRI) in Tehran University of Medical Sciences. The study protocol and the informed consent used for this investigation were approved by the ethical review committees of DDRI and the SUMS. The informed consent form was completed and signed in the presence of other visitors (third party). These forms and the questionnaires are kept in a secure and safe place. The questionnaires are also available with the online version of the article. The project was operated by a 13-member team in Pars Cohort Study Center (PCSC), including general practitioners, nurses, and nutritionists to work on the food frequency questionnaire (FFQ), lab technicians, data entry team, and receptionists in Valashahr and the nearby villages, in southern Fars Province starting in fall, 2012.  This work was funded by Shiraz University of Medical Sciences, Grant Number [910210]. All procedures performed in this study involving human participants were in accordance with the ethical standards of the institutional research committees of both Shiraz University of Medical Sciences and Tehran University of Medical Sciences and with the 1964 Helsinki declaration and its later amendments or comparable ethical standards. Informed consent was obtained from all individual participants included in this study.  <https://pubmed.ncbi.nlm.nih.gov/27349480/> |
| Iran | TLGS | The Tehran Lipid and Glucose Study (TLGS), as the first and longest running cohort of its sort in Iran and the middle east, was initiated in 1999-2001 on a population in district no. 13 of Tehran. The purpose of the study was to determine the prevalence and incidence of risk factors for atherosclerosis among the general urban population of Tehran. It also includes a long-term integrated community-based program for prevention of non-communicable disorders by development of a healthy lifestyle. Totally, 15005 individuals, aged ≥ 3 years, participated in the first examination; re-examinations were conducted in a triennial manner and 3550 individuals were added in the second examination (2002-2005). All participants were also followed up annually and asked about any medical event leading to hospitalization or death. A part of participants was assigned to an educational program for lifestyle modification. The study is still ongoing. Besides the triennial re-examinations during in-person visits, all participants were followed up annually by telephone call to them or their family and asked about any medical event leading to hospitalization during the past year. In case of positive responses, related data were collected by a trained physician using hospital records or, if needed, a home visit. Moreover, in the case of mortality outside the hospital, data were collected from the death certificate, the report of forensic medicine and if needed a verbal autopsy from witnesses. All documents collected were reviewed by an adjudication committee and the final diagnosis was recorded, using a predefined coding protocol. The committee consisted of the physician who collected the data, an internist, an epidemiologist, a cardiologist, an endocrinologist, and other experts invited as needed. Response rate for follow-up was around 80-90 percent for cardiovascular events.  Written informed consent was obtained from all subjects and the study was approved by the ethical committee of Research Institute for Endocrine Sciences.  <https://www.ncbi.nlm.nih.gov/pmc/articles/PMC6289309/>  <https://pubmed.ncbi.nlm.nih.gov/30584434/> |
| Israel | IIHD | The Israeli Ischaemic Heart Disease (IIHD) prospective study was planned to facilitate an approximate representation of mostly migrants from over 20 countries from 5 major areas in 3 continents, Eastern Europe, Central Europe Balkan countries, North African Arab states and Western Asian Mediterranean countries of origin. Diverse sampling rates assured resemblance to ethnic origins of males at ages 40 years through the 65-yr retirement age at the time recruitment began. Over 10,000 male civil servants and municipal employees underwent an extensive clinical, biochemical. Anthropometric, sociodemographic and psychosocial evaluation in the surveys in 1963, 1965 and 1968. Information on use of antihypertensive medications was collected for the 1965 but not in the 1963 survey. Since this information is included as a covariate in the regression models and the 1963 survey is being used as the baseline, those individuals on antihypertensive medications or having no such information available according to the 1965 survey, were excluded from the analyses. They belonged to either government or municipal service in the areas of Tel Aviv, Haifa and Jerusalem. Follow-up for mortality was undertaken in1970 and periodically updated until 2019, primarily based on the national Birth and Mortality Registers. All cancer diagnoses through 2018 were obtained from the National Cancer Registry. Prevalence of Dementia was assessed at 1999/2000 for the remaining 2000 survivors. The original recruitment of patients took place before the establishment of current ethics framework and was arranged through the Israeli Civil Service Commission. Results judged as necessitating added or revised/improved treatment were reported in detail to participants' family physicians. Later end point event assessment was obtained under Helsinki Committee approval or Tel Aviv University Ethics Committee, as appropriate. Informed consent status is unknown. Prime scientific interest, as the study reaches its 60th year of activities in diverse directions, have focused on identified and estimating the role and magnitude of risk factors for the incidence of coronary heart disease, hypertension, subsequently fatal stroke, diagnoses of cancer in specific sites and overall, and survivors' dementia.  <https://pubmed.ncbi.nlm.nih.gov/8324774/>  <https://pubmed.ncbi.nlm.nih.gov/15308782/> |

**Central, East and South Asia**

| Country | Study | Study/cohort full name and short description |
| --- | --- | --- |
| Brunei | WHO STEPS Brunei | The Brunei STEPS cohort study is a national health survey conducted in 2015. It is based on the WHO STEPwise methodology for surveillance (STEPS) of NCDs and risk factors which is an internationally comparable, standardized and integrated surveillance tool through which countries can collect, analyse and disseminate core information on noncommunicable diseases (NCDs). The selection criteria for the study were Brunei citizens or permanent residents aged 18 to 69 years with regular stay throughout the year, not pregnant or diagnosed with terminal or incapacitating illnesses. A total of 3808 individuals participated in the step 1 questionnaire interview, 2082 participated in steps 2 anthropometric and BP measurements as well as biochemistry tests. Survey data were supplemented with clinical information from the national electronic medical record system for cause-specific mortality based on ICD-10 codes. Data available include sociodemographic profiles, tobacco use, alcohol consumption, dietary habits, the amount of physical activity (PA), history of cardiovascular diseases, height, weight, waist circumference and BP measurements, fasting blood sugar and cholesterol levels, and spot urine sodium.  Ethics approval of the study was granted through the Medical and Health Research Ethics Committee, Ministry of Health Brunei Darussalam, prior to data collection (MHREC/MOH/2015/11(9)).  References:  WHO STEPS 2015 Brunei Darussalam NCD Microdata Repository. Available at <https://extranet.who.int/ncdsmicrodata/index.php/catalog/494>  Ong, S. K. et al. “Cross-sectional STEPwise Approach to Surveillance (STEPS) Population Survey of Noncommunicable Diseases (NCDs) and Risk Factors in Brunei Darussalam 2016.” Asia-Pacific journal of public health vol. 29,8 (2017): 635-648. Doi:10.1177/1010539517738072  Ong S. K. et al. Prevalence of undetected hypertension and its association with socio-demographic and non-communicable diseases risk factors in Brunei Darussalam. Journal of Public Health; Heidelberg Vol. 31, Iss. 1, (Jan 2023): 149-160. Doi:10.1007/s10389-020-01287-y |
| Japan | Hisayama | The Hisayama Study is a population-based prospective cohort study that has been conducted in the town of Hisayama, Japan since 1961 and is still ongoing. The town of Hisayama is located in a suburb of the Fukuoka metropolitan area in Kyushu, Japan. This study is designed to evaluate the risk factors for lifestyle-related diseases, such as stroke, coronary heart disease, hypertension, diabetes, and dementia, in a general Japanese population.  For this collaboration the individual participant data from the cohort in 1988-2012, which includes a total of 2742 residents aged ≥40 years was sent. 80.9% of the total population in this age group were followed up annually prospectively for 24 years, from December 1988 to November 2012, via surveys. All available information about potential CVD events and deaths among the study participants was collected and reviewed by physician members of the study to determine the occurrence of CVD events or cause of death under the standardized diagnostic criteria throughout the study period. Furthermore, if consent for an autopsy was given, autopsy findings were used to adjudicate the underlying cause of death and confirm the existence of CVD (stroke lesions, myocardial necrosis, and atherosclerotic lesions in coronary, carotid, cerebral, and other major arteries) and to classify subtypes of stroke.  The ethics committee of Kyushu University approved this study, participants provided written informed consent, and the procedures followed were in accordance with national guidelines.  <https://pubmed.ncbi.nlm.nih.gov/30298863/> <https://pubmed.ncbi.nlm.nih.gov/23902756/> |
| Japan | Yamagata | The Yamagata Study is a community-based study and recruited its participants older than 40 years between 2009 and 2015, who received an annual health check in the Yamagata Prefecture. The aim of Yamagata Study is to elucidate the pathology of risk genes targeting 5 major cancers (lung cancer, stomach cancer, colon cancer, liver cancer (hepatitis), breast cancer), stroke, acute myocardial infarction, hypertension, renal failure, and diabetes, and further to identify drug targets. Hypertension at baseline was defined as a systolic blood pressure ≥140 mm Hg or diastolic blood pressure ≥90 mm Hg, or the use of antihypertensive medications. Subjects with a baseline body-mass index ≥25.0 kg/m^2^ were categorized as obese. Presence of diabetes was defined as a plasma glucose level ≥126 mg/dL, hemoglobin A1c ≥6.5% (Japanese Diabetes Society value), or the use of antidiabetic medications. Incidence was followed up through the linkage with the Yamagata Stroke and Acute Myocardial Infarction (AMI) registries and the Yamagata Society in Treatment for Cerebral Stroke (YSTCS). Fatal events were collected through ICD-10 coding from causes of death of death certificates.  This analysis included 20,971 subjects that were followed up until end of 2016. All subjects provided written informed consent prior to enrolment.  The Yamagata study is supported in part by a Grant in-Aid from the 21st Century Center of Excellence (COE) and Global COE program of the Japan Society for the Promotion of Science.  <https://www.ncbi.nlm.nih.gov/pmc/articles/PMC7064551/> |
| Nepal | KHDC | KHDC started as a community-based initiative where adult population, above 18 years of age, residing in and around Dharan municipality of Eastern Nepal. The focus of initiative was primary prevention of leading NCDs through community awareness on lifestyle modification with; weight control (diet modification and physical activity), and tobacco cessation. Concurrently, provisions for secondary prevention through control of blood pressure, blood and urine analysis, were undertaken through mass screening campaigns. This cohort includes population-level data from individuals assessed during a period 2007-2019. The screening initiative started as a voluntary service led by Department of Internal Medicine at BP Koirala Institute of Health Sciences, a publicly funded tertiary care centre in the region. The cohort were assessed for sociodemographic profiles, family and personal medical history, dietary habit, physical activity, tobacco/alcohol consumption, body mass index, hip/waist circumference, blood pressure measurement, fasting blood sugar, serum creatinine, lipid profile, proteinuria and consultation services. Consultation services were provided to the cases diagnosed to prevent disease progression and advocated for treatment adherence through regular follow up. This cohort includes 28,326 (KHDC 1: 17082; KHDC 2: 11244) participant data with follow up information on mortality, occurrence of selected NCDs, and complications including stroke, heart failure and renal failure. Mortality information was obtained through community health worker follow-up during regular NCD screenings and death certificates where available. The cause of death was reviewed by a physician review process.  Informed consent was obtained from all participants prior to inclusion.  Sharma SK, Zou H, Togtokh A, Ene-Iordache B, Carminati S, Remuzzi A, Wiebe N, Ayyalasomayajula B, Perico N, Remuzzi G, Tonelli M. Burden of CKD, proteinuria, and cardiovascular risk among Chinese, Mongolian, and Nepalese participants in the International Society of Nephrology screening programs. Am J Kidney Dis 2010; 56(5):915-27.  Sharma SK, Ghimire A, Radhakrishnan J, Thapa L, Shrestha NR, Paudel N, Gurung K, R M, Budathoki A, Baral N, Brodie D. Prevalence of hypertension, obesity, diabetes, and metabolic syndrome in Nepal. Int J Hypertens 2011; 2011:821971 |

## Description of health examination survey data

**Europe**

| Country | Study | Study/cohort full name and short description |
| --- | --- | --- |
| Germany | DEGS1 | The German Health Interview and Examination Survey for Adults (DEGS1, Studie zur Gesundheit Erwachsener in Deutschland) is a randomly chosen national sample of the adult population in Germany age between 18-79 years. Between 2008 and 2011 DEGS1 recruited 8,152 subjects (4,193 newly recruited participants with a response rate of 42% and 3,959 who had previously taken part in GNHIES98 with a response rate of 62%). It aims to provide current and normative data for the clinical research and investigate the health-related quality of life in Germany. The survey includes a visit to a local study center for examinations and a questionnaire for the survey. DEGS1 was approved by the local IRB and participants prove informed consent.  <https://www.rki.de/EN/Content/Health_Monitoring/HealthSurveys/Degs/degs_content.html>  <https://pubmed.ncbi.nlm.nih.gov/22938722/>  Robert Koch-Institut, Abteilung für Epidemiologie und Gesundheitsmonitoring (2015): Studie zur Gesundheit Erwachsener in Deutschland (DEGS1). Scientific Use File 1. Version.  <https://doi.org/10.7797/16-200812-1-1-1> |

| Azerbaijan | WHO STEPS Azerbaijan | The WHO STEPS survey Azerbaijan is a population-based survey that was carried out in 2017 and included a total of 1852 households from 240 regional clusters and included participants age 18-69 years. A multi-stage cluster sampling design was used to produce representative data for that age range in Azerbaijan. The survey collected data on socio-demographics, medical history, behavioural information, and physical and biochemical measurements. 2880 individual adults were included in this survey.  <https://extranet.who.int/ncdsmicrodata/index.php/catalog/127> |
| --- | --- | --- |
| Belarus | WHO STEPS  Belarus | The WHO STEPS survey Belarus is a population-based survey that was carried out between 2016 and 2017 and included a total of 5760 participants aged 18-69. A Multi-stage cluster sampling design was used to produce representative data for that age range in Belarus. The overall response rate was 87.1%. The survey collected data on socio-demographics, medical history, behavioural information, and physical and biochemical measurements.  <https://extranet.who.int/ncdsmicrodata/index.php/catalog/100> |
| Georgia | WHO STEPS  Georgia | The WHO STEPS survey Georgia was established in 2010 and is a population-based survey of adults age 18-69 years. For this analysis the second STEPS survey from 2016 was used. A multi-stage cluster sampling design was used to produce representative data for that age range in Georgia. A total of 5554 adults were included in the survey. The overall response rate was 75.7%. The survey collected data on socio-demographics, medical history, behavioural information, and physical and biochemical measurements.  <https://extranet.who.int/ncdsmicrodata/index.php/catalog/223> |
| Moldova | WHO STEPS Moldova | The WHO STEPS Moldova survey was established in 2013 and is a population-based survey of adults 18-69. A multi-stage cluster sampling design was used to produce representative data for that age range in Georgia. A total of 4807 adults were included in the survey. The overall response rate was 83.5%. The survey collected data on socio-demographics, medical history, behavioural information, and physical and biochemical measurements.  <https://extranet.who.int/ncdsmicrodata/index.php/catalog/230> |
| Tajikistan | WHO STEPS Tajikistan | The WHO STEPS survey Tajikistan was established in 2016 and is a population-based survey of adults age 18-69 years. For this analysis the STEPS survey from 2016/2017 was used. A multi-stage cluster sampling design was used to produce representative data for that age range in Tajikistan. A total of 2718 adults were included in the survey. The overall response rate was 94.3%. The survey collected data on socio-demographics, medical history, behavioural information, and physical and biochemical measurements.  <https://extranet.who.int/ncdsmicrodata/index.php/catalog/270> |
| Turk-menistan | WHO STEPS Turk-menistan | The WHO STEPS survey Turkmenistan was established in 2013 and is a population-based survey of adults age 18-69 years. For this analysis the second STEPS survey from 2018 was used. A multi-stage cluster sampling design was used to produce representative data for that age range in Turkmenistan. A total of 4053 adults were included in the survey. The overall response rate was 93.8%. The survey collected data on socio-demographics, medical history, behavioural information, and physical and biochemical measurements.  <https://extranet.who.int/ncdsmicrodata/index.php/catalog/854> |

**The Western Asia and North Africa**

| Country | Study | Study/cohort full name and short description |
| --- | --- | --- |
| Iraq | WHO STEPS Iraq | The WHO STEPS survey Iraq was established in 2006 and is a population-based survey of adults age 18 years or older. For this analysis the second STEPS survey from 2015 was used. A multi-stage cluster sampling design was used to produce representative data for that age range in Iraq. A total of 4120 adults were included in the survey. The overall response rate was 93.5%. The survey collected data on socio-demographics, medical history, behavioural information, and physical and biochemical measurements. Additionally, the survey also tested the concentration of salt in urine samples.  <https://extranet.who.int/ncdsmicrodata/index.php/catalog/420> |
| Jordan | WHO STEPS Jordan | The WHO STEPS survey Jordan was established in 2007 and is a population-based survey of adults age 18-69 years. For this analysis the second STEPS survey from 2019 was used. A multi-stage cluster sampling design was used to produce representative data for that age range in Jordan. A total of 5713 adults were included in the survey. The response rate was 63%. The survey collected data on socio-demographics, medical history, behavioural information, and physical and biochemical measurements.  <https://extranet.who.int/ncdsmicrodata/index.php/catalog/853> |
| Lebanon | WHO STEPS Lebanon | The WHO STEPS survey Lebanon was established in 2016 and is a population-based survey of adults age 18-69 years. A multi-stage cluster sampling design was used to produce representative data for that age range in Lebanon. As Lebanon has a significant number of Syrian immigrants and refugees, a Lebanese (n=1899) and a Syrian sample (n=2134), was created. For this analysis both STEPS survey samples from 2017 were used. A total of 4033 adults were included in the survey. The overall response rate for the Lebanese sample was 65.9% and 74.1% for the Syrian sample. The survey collected data on socio-demographics, medical history, behavioural information, and physical and biochemical measurements.  <https://extranet.who.int/ncdsmicrodata/index.php/catalog/410> |
| Morocco | WHO STEPS Morocco | The WHO STEPS survey Morocco was established in 2017 and is a population-based survey of adults aged 18 years and older. A multi-stage cluster sampling design was used to produce representative data for that age range in Sudan. A total of 5429 adults were included in the survey. The overall response rate was 89%. The survey collected data on socio-demographics, medical history, behavioural information, and physical and biochemical measurements.  <https://extranet.who.int/ncdsmicrodata/index.php/catalog/544> |
| Qatar | WHO STEPS Qatar | The WHO STEPS survey Qatar was established in 2017 and is a population-based survey of adults aged 18-64 years. A multi-stage cluster sampling design was used to produce representative data for that age range in Sudan. A total of 2496 adults were included in the survey. The overall response rate was 88%. The survey collected data on socio-demographics, medical history, behavioural information, and physical and biochemical measurements.  <https://extranet.who.int/ncdsmicrodata/index.php/catalog/708> |
| Sudan | WHO STEPS Sudan | The WHO STEPS survey Sudan was established in 2016 and is a population-based survey of adults age 18-69 years. For this analysis the STEPS survey from 2016 was used. A multi-stage cluster sampling design was used to produce representative data for that age range in Sudan. A total of 8145 adults were included in the survey. The overall response rate was 95%. The survey collected data on socio-demographics, medical history, behavioural information, and physical and biochemical measurements.  <https://extranet.who.int/ncdsmicrodata/index.php/catalog/438> |

**Central, East and South Asia**

| Country | Study | Study/cohort full name and short description |
| --- | --- | --- |
| Bangladesh | WHO STEPS Bangladesh | The WHO STEPS survey Bangladesh was established in 2009 and is a population-based survey of adults age 18-69 years. For this analysis the second STEPS survey from 2018 was used. A multi-stage cluster sampling design was used to produce representative data for that age range in Bangladesh. A total of 8185 adults were included in the survey. The overall response rate was around 80%. The survey collected data on socio-demographics, medical history, behavioural information, and physical and biochemical measurements. Additionally, the survey collected data on oral health and an assessment of the national health system and its ability to detect and treat key risk factors for non-communicable diseases.  <https://extranet.who.int/ncdsmicrodata/index.php/catalog/770> |
| Bhutan | WHO STEPS Bhutan | The WHO STEPS survey Bhutan was established in 2014 and is a population-based survey of adults age 15-6 years. For this analysis the second STEPS survey from 2019 was used. A multi-stage cluster sampling design was used to produce representative data for that age range in Bhutan. A total of 5575 adults were included in the survey. The overall response rate was around 94.8%. The survey collected data on socio-demographics, medical history, behavioural information, and physical and biochemical measurements.  [https://extranet.who.int/ncdsmicrodata/index.php/catalog/855](https://extranet.who.int/ncdsmicrodata/index.php/catalog/855/download/6026) |
| Korea | KNHANES | The Korea National Health and Nutrition Examination Survey (KNHANES) is a national surveillance system assessing the health and nutritional status of the Korean population since 1998. The 2011 survey included 8,518 participants aged ≥1 year; for the present analyses, only adults aged ≥18 years were included (n = 7,816).  KNHANES collects demographic, social, health, and nutritional data through three components: a health interview, health examination, and nutrition survey. The health interview and examination are conducted at mobile examination centers across the country, while the nutrition survey is conducted in participants’ homes approximately one week later.  The health interview includes both household- and individual-level components. Household information, including demographics and income, is provided by an adult respondent. The individual component captures lifestyle factors (e.g., smoking, alcohol use, physical activity), health status, and healthcare utilization through a combination of self-administered questionnaires and face-to-face interviews, with content varying by age.  All participants used in the analyses able to provide informed consent, did provide informed consent.  https://academic.oup.com/ije/article/43/1/69/733063 |
| Lao | WHO STEPS Lao | The WHO STEPS survey Lao was established in 2013 and is a population-based survey of adults age 18-84 years. A multi-stage cluster sampling design was used to produce representative data for that age range in Bhutan. The overall response rate was around 99.2%. The survey collected data on socio-demographics, medical history, behavioural information, and physical and biochemical measurements.  <https://extranet.who.int/ncdsmicrodata/index.php/catalog/588> |
| Mongolia | WHO STEPS Mongolia | The WHO STEPS survey Mongolia was established in 2019 and is a population-based survey of adults age 15-6 years. A multi-stage cluster sampling design was used to produce representative data for that age range in Bhutan. A total of 6654 adults were included in the survey. The overall response rate was around 98.1%. The survey collected data on socio-demographics, medical history, behavioural information, and physical and biochemical measurements.  <https://extranet.who.int/ncdsmicrodata/index.php/catalog/836> |
| Myanmar | WHO STEPS Myanmar | The WHO STEPS survey Myanmar was carried out from September to December in 2014. The survey was a population-based survey of adults age 25-64 years. A multi-stage cluster sample design was used to produce representative data for that age range from 52 townships in Myanmar. A total of 8757 adults participated in the survey. The survey collected information on sociodemographic and behavioural characteristics, physical measurements such as height, weight and blood pressure and biochemical measurements (blood glucose-both fasting and 2-h PG, levels of total cholesterol, triglycerides, HDL and LDL. The overall response rate was 94% for risk factors, 91% for physical measurements and 90% for biochemical measurements.  <https://www.who.int/publications/m/item/2014-steps-country-report-myanmar> |

# Supplementary results

## Supplementary figures

## Figure S1: Study flow chart

**Eurasian C&C Risk collaboration**

Harmonized dataset

**Longitudinal population studies**

N = 798,867 individuals, aged 18-100 years
N = 25 cohorts
N = 17 countries

**Cross-sectional health examination surveys (HES)**

N = 84,380 individuals representing a total of 303,435,348 inhabitants

N = 19 cohorts
N = 18 countries

Excluded data

- 101,700 with prevalent cardiovascular disease or cancer
- 27,418 with missing information on cardiovascular or cancer mortality
- 15,724 with missing covariates after multiple imputation

*Available for analyses*

**Main Analysis**

Five modifiable risk factors for CVD and cancer mortality

N = 670,304

Excluded data

- 74,156 individuals with missing data on education after multiple imputation

Secondary Analysis

Accounting for years of completed education

N = 580,424

## Figure S2: Analytical workflow

This figure depicts the analytical workflow beginning with the transferred individual-level data from an individual cohort to the different analyses undertaken. Abbreviations: HR, Hazard Ratio; PAF, population-attributable fractions

Figure 3a: Cumulative incidences for cardiovascular mortality (red lines) and cancer mortality (blue lines) using age as time scale by sex.
Individuals with known cardiovascular disease or cancer at baseline were excluded. Curves are shown for an index age of 50 years.

**Women**

**Men**

Figure S3b: Cumulative incidences for cardiovascular mortality (red lines) and cancer mortality (blue lines) using time since study entry as scale by sex
Individuals with known cardiovascular disease or cancer at baseline were excluded. Curves are shown for an index age of 50 years. Follow-up duration in years was used as time scale.

**Women**

**Men**

## Figure S4: Cumulative incidences for cardiovascular mortality (red lines) and cancer mortality (blue lines) using age as time scale a) with UK Biobank and b) without UK Biobank by sex.

Individuals with known cardiovascular disease or cancer at baseline were excluded. Curves are shown for an index age of 50 years.

**Women**

**Men**

## Figure S5: Associations of risk factors with cardiovascular (red lines) and cancer mortality (blue lines) allowing for effects to change with age by sex.

Shown are the results of a regional 1-year landmark analysis that allowed for effects to change with age. Age was used as the time scale. All five risk factors considered were included in the Cox proportional hazards models together with use of antihypertensive medications. Regional estimates are presented. HR for systolic BP is shown per 20mmHg, for Non-HDL cholesterol per 1mmol/L, for BMI per 5kg/m^2^. Abbreviations: BP, blood pressure; non-HDL, non-high-density lipoprotein cholesterol; BMI, body-mass index.

**Women**

**Men**

## Figure S6: Risk factor associations for cardiovascular and cancer mortality from linear Cox models by region and sex.

**S**hown are the results of a 1-year landmark analysis for linear effects. Age was used as the time scale. All five risk factors, together with the use of antihypertensive medications, were included as covariates in the Cox proportional hazards models. Individuals with cardiovascular disease or cancer at baseline were excluded. Abbreviations: BP, blood pressure; non-HDL, non-high-density lipoprotein cholesterol; BMI, body-mass index.

**Women**

**Men**

## Figure S7: Risk factor associations with cardiovascular disease (red) and cancer mortality (blue) including education by region and sex.

Shown are the results of a 1-year landmark analysis allowing for nonlinear effects. Age was used as the time scale. All five risk factors, together with the use of antihypertensive medications, were included as covariates in the Cox proportional hazards models. Continuous risk factors were modelled using cubic splines. The widths of the 95% confidence intervals are displayed as shaded areas. The p-value shown is for a test for the equality of the covariate's coefficients across the two endpoints. Abbreviations: non-HDL, non-high-density lipoprotein.

**Women**

**Men**

## Figure S8: Risk factor associations for non-cardiovascular and non-cancer mortality from linear Cox models by sex and region.

**S**hown are the results of a 1-year landmark analysis for linear effects. Age was used as the time scale. All five risk factors, together with the use of antihypertensive medications, were included as covariates in the Cox proportional hazards models. Individuals with cardiovascular disease or cancer at baseline were excluded. Abbreviations: BP, blood pressure; non-HDL, non-high-density lipoprotein cholesterol; BMI, body-mass index.

**Women**

**Men**

## Figure S9: Risk factor associations with cardiovascular disease (red) and cancer mortality (blue) adjusting for examination year, by sex and year.

Shown are the results of a 1-year landmark analysis allowing for nonlinear effects. Age was used as the time scale. All five risk factors, together with the use of antihypertensive medications and year of examination, were included as covariates in the Cox proportional hazards models. Continuous risk factors were modelled using cubic splines. The widths of the 95% confidence intervals are displayed as shaded areas. The p-value shown is for a test for the equality of the covariate's coefficients across the two endpoints. Abbreviations: non-HDL, non-high-density lipoprotein.

**Women**

**Men**

## Figure S10: Risk factor associations for cardiovascular and cancer mortality from linear Cox models by sex and region, adjusting for examination year.

**S**hown are the results of a 1-year landmark analysis for linear effects. Age was used as the time scale. All five risk factors, together with the use of antihypertensive medications´ and year of examination, were included as covariates in the Cox proportional hazards models. Individuals with cardiovascular disease or cancer at baseline were excluded. Abbreviations: BP, blood pressure; non-HDL, non-high-density lipoprotein cholesterol; BMI, body-mass index.

**Women**

**Men**

## Figure S11: Risk factor associations for cardiovascular and cancer mortality from linear Cox models, by region, in cohorts recruited after 1988.

**S**hown are the results of a 1-year landmark analysis for linear effects. Age was used as the time scale. All five risk factors, together with the use of antihypertensive medications´ and year of examination, were included as exposures in the Cox proportional hazards models. Individuals with cardiovascular disease or cancer at baseline or recruitment year before 1988 were excluded. Abbreviations: CVM, cardiovascular mortality; CM, cancer mortality; BP, blood pressure; non-HDL, non-high-density lipoprotein cholesterol; BMI, body-mass index.

**Women**

**Men**

## Figure S12: Population-attributable fractions for cardiovascular and cancer mortality, excluding the UK Biobank by region and sex

Population-attributable fractions were estimated with the use of Weibull regression models. PAFs for single risk factors and aggregated PAF for the risk factors combined are shown.
Abbreviations: SBP, systolic blood pressure; non-HDL-C, non-high-density lipoprotein cholesterol; BMI, body-mass index.

## Figure S13: Unadjusted population-attributable fractions for cardiovascular and cancer mortality

Population-attributable fractions were estimated with the use of Weibull regression models. PAFs for single risk factors are shown without adjustment for any of the other exposures.
Abbreviations: SBP, systolic blood pressure; non-HDL-C, non-high-density lipoprotein cholesterol; BMI, body-mass index.

## Figure S14: A) Joint population-attributable fractions of five risk factors for 10-year incidence of cardiovascular and cancer mortality by sex and region, and B) Current healthcare expenditure per capita and region.

Population-attributable fractions (PAF) were estimated with the use of Weibull regression models. Joint PAF for the risk factors combined are shown. Current health expenditure per capita was based on national WHO data and pooled for the given regions. Abbreviations: PAF, population-attributable fractions.

B

A

## Figure S15: Population-attributable fractions of modifiable risk factors and education for cardiovascular and cancer mortality by geographic region and sex

Population-attributable fractions were estimated with the use of Weibull regression models. PAFs for single risk factors and aggregated PAF for the risk factors combined are shown.
Abbreviations: SBP, systolic blood pressure; non-HDL-C, non-high-density lipoprotein cholesterol; BMI, body-mass index.

## Figure S16: Population-attributable fractions of modifiable risk factors for premature cardiovascular and cancer mortality by geographic region and sex

Population-attributable fractions were estimated with the use of Weibull regression models. PAFs for single risk factors and aggregated PAF for the risk factors combined are shown.
Abbreviations: SBP, systolic blood pressure; non-HDL-C, non-high-density lipoprotein cholesterol; BMI, body-mass index.

## Supplementary tables

| Table S1: STROBE statement for cohort studies - completed checklist. | | | | |
| --- | --- | --- | --- | --- |
|  | Item No | Recommendation | Page No. | Relevant text from manuscript |
| **Title and abstract** | 1 | (a) Indicate the study’s design with a commonly used term in the title or the abstract | 1 | See title |
|  |  | (b) Provide in the abstract an informative and balanced summary of what was done and what was found | 2 | See abstract |
| Introduction | | |  |  |
| Background/rationale | 2 | Explain the scientific background and rationale for the investigation being reported | 6 |  |
| Objectives | 3 | State specific objectives, including any prespecified hypotheses | 6 |  |
| Methods | | |  |  |
| Study design | 4 | Present key elements of study design early in the paper | 7 |  |
| Setting | 5 | Describe the setting, locations, and relevant dates, including periods of recruitment, exposure, follow-up, and data collection | 7 |  |
| Participants | 6 | (a) Give the eligibility criteria, and the sources and methods of selection of participants. Describe methods of follow-up | 7 |  |
|  |  | (b) For matched studies, give matching criteria and number of exposed and unexposed |  |  |
| Variables | 7 | Clearly define all outcomes, exposures, predictors, potential confounders, and effect modifiers. Give diagnostic criteria, if applicable | 7 | Supplement Tables S2-S4 |
| Data sources/ measurement | 8* | For each variable of interest, give sources of data and details of methods of assessment (measurement). Describe comparability of assessment methods if there is more than one group | 7 | Supplement Tables S2-S4 |
| Bias | 9 | Describe any efforts to address potential sources of bias | 12-13 | Limitations section |
| Study size | 10 | Explain how the study size was arrived at |  | Supplement Figure S1 |
| Quantitative variables | 11 | Explain how quantitative variables were handled in the analyses. If applicable, describe which groupings were chosen and why | 6-7 |  |
| Statistical methods | 12 | (a) Describe all statistical methods, including those used to control for confounding | 6-7 |  |
|  |  | (b) Describe any methods used to examine subgroups and interactions | 6-7 |  |
|  |  | (c) Explain how missing data were addressed | 6 | Line 12 |
|  |  | (d) If applicable, explain how loss to follow-up was addressed |  |  |
|  |  | (e) Describe any sensitivity analyses | 7 | Line 17-22 |
| Results | | |  |  |
| Participants | 13* | (a) Report numbers of individuals at each stage of study—eg numbers potentially eligible, examined for eligibility, confirmed eligible, included in the study, completing follow-up, and analysed |  | Supplement Figure S1 |
|  |  | (b) Give reasons for non-participation at each stage |  | Supplement Figure S1 |
|  |  | (c) Consider use of a flow diagram |  | Supplement Figure S1 |
| Descriptive data | 14* | (a) Give characteristics of study participants (eg demographic, clinical, social) and information on exposures and potential confounders | 5 | Line 2-17 |
|  |  | (b) Indicate number of participants with missing data for each variable of interest |  | Supplement Table S4 |
|  |  | (c) Summarise follow-up time (eg, average and total amount) |  | Supplement Table 7 |
| Outcome data | 15* | Report numbers of outcome events or summary measures over time |  | Supplement Table 6a &b |
| Main results | 16 | (a) Give unadjusted estimates and, if applicable, confounder-adjusted estimates and their precision (eg, 95% confidence interval). Make clear which confounders were adjusted for and why they were included |  |  |
|  |  | (b) Report category boundaries when continuous variables were categorized |  | Supplement Table S8, Table S10, Table S12, Table S14 |
|  |  | (c) If relevant, consider translating estimates of relative risk into absolute risk for a meaningful time period |  |  |
| Other analyses | 17 | Report other analyses done—eg analyses of subgroups and interactions, and sensitivity analyses |  | Supplement Figure S3, Figure S8-S9 |
| Discussion | | |  |  |
| Key results | 18 | Summarise key results with reference to study objectives | 13 | Line 3-10 |
| Limitations | 19 | Discuss limitations of the study, taking into account sources of potential bias or imprecision. Discuss both direction and magnitude of any potential bias | 15-16 |  |
| Interpretation | 20 | Give a cautious overall interpretation of results considering objectives, limitations, multiplicity of analyses, results from similar studies, and other relevant evidence | 14-15 |  |
| Generalisability | 21 | Discuss the generalisability (external validity) of the study results | 14 | Line 13-14 |
| Other information | | |  |  |
| Funding | 22 | Give the source of funding and the role of the funders for the present study and, if applicable, for the original study on which the present article is based | 17 |  |

| Table S2: Core variables of interest | |
| --- | --- |
| **Variable** | **Description** |
| Age | Participant’s age at baseline |
| Sex | Participant’s sex |
| Body-mass index | Participant’s measured body-mass index at baseline |
| Current smoking | Self-reported daily or occasional tobacco consumption (including cigars, cigarillos or pipes, and excluding e-cigarettes) |
| Education | Years of completed education |
| Diabetes | Self-reported or diagnosed history of diabetes |
| Systolic blood pressure | Participant’s measured systolic blood pressure (mean of the second and third measurement) |
| Diastolic blood pressure | Participant’s measured diastolic blood pressure (mean of the second and third measurement) |
| Antihypertensive medication | Intake of antihypertensive medication (self-reported/medical records) |
| Total cholesterol | Participant’s measured total cholesterol |
| Non-HDL cholesterol | Participant’s derived non-HDL cholesterol |
| HDL cholesterol | Participant’s measured HDL cholesterol |
| Lipid-lowering medication | Intake of lipid-lowering medication (self-reported/medical records) |
| History of cardiovascular disease | Any history of coronary heart disease, unstable angina, myocardial infarction, previous coronary revascularization or stroke at baseline (medical records) |
| History of cancer | Any documented history of cancer excluding melanoma |
| CVD mortality | Death due to an cardiovascular event or sudden cardiac arrest |
| Cancer mortality | Death due to any cancer |
| Premature death | Any death occurring before the age of 70 |

| **Table S3: End point definitions** | | | |
| --- | --- | --- | --- |
| Eurasian C&C Risk collaboration definitions | **Definition and composition** | **Corresponding ICD code** | |
|  |  | **ICD-10** | **ICD-9** |
| Cardiovascular Disease |  |  |  |
| CHD death | Fatal CHD event, cardiac arrest or unclassifiable death | I20.0-I25, I46, R96, R98, R99 | 410-414, 798 |
| Stroke death | Death due to cerebrovascular event | I60-I64, I69 | 430-434,  436, 438 |
| Cancer |  |  |  |
| Cancer death | Fatal cancer event | C00–C97 | 140–208 |

| Table S4: Variable Definitions and Coding | | |
| --- | --- | --- |
| **Variable** | **Description & coding** | **Unit** |
| **Baseline variables** | | |
| Date of Baseline Examination | If the person attended the survey examination, enter here the exact date when the physical/clinical examination of the person was carried out. If the person comes to the clinic more than once enter here the date which best identifies the day of the risk factor assessments (smoking, blood pressure, venous blood specimen taken for cholesterol).  If the person returns the completed questionnaire by mail but does not come to the clinic and has left this item blank, enter here the date of return of the mailed questionnaire.  Please make sure that all dates correspond with the study period in your centre. | DDMMYYYY  (day,  month,  year) |
| Age | Derived age at the date of examination. AGE1 is calculated as a difference between the date of birth and the date of examination. | years  (2 decimals) |
| Sex | - 1 = Male - 2 = Female |  |
| BMI | 99.99 = insufficient data  BMI to two decimal places in kg/m², derived from items HEIGHT (in cm) and WEIGHT (in kg) as:  BMI = WEIGHT / (HEIGHT / 100)²  The result is rounded to the nearest two decimals. | kg/m²  (2 decimals) |
| Smoking | Current smoker of cigarettes, cigars/cigarillos or pipe  1 = yes  2 = no  9 = insufficient data |  |
| Systolic Blood Pressure | Systolic blood pressure, mean of two measurements. Note that when only one measurement value is available, that is used.   - 999.9 = insufficient data | mmHg  (1 decimal) |
| Diastolic Blood Pressure | Diastolic blood pressure, mean of two measurements. Note that when only one measurement value is available, that is used.   - 999.9 = insufficient data | mmHg  (1 decimal) |
| Antihypertensive Medication | Taking antihypertensive drugs   - 1 = yes - 2 = no - 9 = insufficient data   Based on ATC medication categories  C02 = antihypertensives   - 1 = yes - 2 = no - 9 = insufficient data |  |
| Diabetes | Documented or self-reported history of diabetes   - 1 = yes - 2 = no - 9 = insufficient data   This history item is defined for excluding prevalent cases for incident diabetes.  BASEDIAB1 includes both type 1 and type 2 diabetes. |  |
| Cancer | Documented or self-reported history of cancer   - 1 = yes - 2 = no - 9 = insufficient data |  |
| Heart Attack | Documented or self-reported history of MI, including angina pectoris when the data does not permit its separation from MI   - 1 = yes - 2 = no   9 = insufficient data |  |
| Cardiac Revascularization | Documented or self-reported cardiac revascularization   - 1 = yes - 2 = no - 9 = insufficient data   The purpose of this item is to indicate the information on previous cardiac revascularization i.e. coronary artery bypass (CABG) or percutaneous transluminal coronary angioplasty (PTCA) at baseline. |  |
| Stroke | Documented or self-reported history of stroke   - 1=yes - 2=no - 9=insufficient data |  |
| Cardiovascular Disease | Documented or self-reported history of MI or stroke, including angina pectoris when the data does not permit its separation from MI - 1 = yes - 2 = no - 9 = insufficient data Cardiovascular Disease is derived from items heart attack and stroke as: \| \| Heart Attack \| \|; \| \| 1 \| 2 \| 9; Stroke \| 1 \| Cardiovascular Disease = 1 \| Cardiovascular Disease = 1 \| Cardiovascular Disease = 1; \| 2 \| Cardiovascular Disease = 1 \| Cardiovascular Disease = 2 \| Cardiovascular Disease = 9; \| 9 \| Cardiovascular Disease = 1 \| Cardiovascular Disease = 9 \| Cardiovascular Disease = 9 |  |
| Total Cholesterol | Total serum cholesterol   - 99.99 = insufficient data |  |
| HDL cholesterol | HDL serum cholesterol   - 9.999 = insufficient data |  |
| Calculated LDL Cholesterol | LDL cholesterol to two decimal places. LDL is calculated from the Friedewald formula which is considered to be invalid if serum triglycerides > 4.52 mmol/l or there was less than 12h fasting prior to blood sampling. |  |
| Measured LDL cholesterol | Directly measured LDL cholesterol |  |
| Triglycerides | Serum triglycerides   - 99.99 = insufficient data |  |
| Lipid-lowering Medication | Taking drugs for lowering cholesterol levels   - 1 = yes - 2 = no - 9 = insufficient data   Based on ATC medication categories  C10 = lipid modifying agents   - 1 = yes - 2 = no - 9 = insufficient data |  |
| Fasting Status | Fasting status before blood sampling   - 1 = overnight fasting or fasting known to have lasted at least 8 hours - 2 = fasting before blood sampling lasted at least 4 hours but less than overnight or 8 hours - 3 = the subject has eaten within 4 hours before blood sampling - 9 = insufficient data |  |
| **Follow-up variables** | | |
| Death Status | Death due to any cause   - 1 = if the person died during the follow-up. - 2 = if the person did not die during the follow-up. - 9 = when there are no follow-up data available due to any reason.   The follow-up starts at the date of baseline examination. |  |
| Date of Death | Date of exit from the mortality follow-up. The individual's follow-up period ends at his exit from the study. The exit takes place when the earliest one of the following events occur:   (a) the person dies; (b) the fixed follow-up period of the cohort for the study ends; (c) the person refuses from taking part in the study; (d) the person is lost to follow-up, i.e. it is no longer possible to follow-up the person for his or her death with reasonable efforts. | DDMMYYYY  (day,  month,  year) |
| Time to Death | Length of mortality follow-up. It is the difference between the date of death and date of examination | days |
| Age at Death | Age at the time of exit from the mortality follow-up. It is the sum of the age at the examination and time to death divided by 365.25. | Years |
| Coronary Death | Coronary death  • 1 = yes  • 2 = no coronary death during follow-up  • 9 = insufficient data  Coronary death is coded 1 if the person died due to coronary heart disease or from unknown cause during the follow-up.  Coronary death is coded 2 if the person did not die due to coronary heart disease or from unknown cause during the follow-up.  Coronary death is coded 9 when there are no follow-up data available due to any reason.  Note that coronary death = 2 includes those who survived during the follow-up or who died due to known cause other than coronary heart disease. For the analysis of coronary death as an endpoint, the date, follow-up time and age at the time of exit from the follow-up of coronary death are the same as date of death, time to death and age at death. | ^ |
| Stroke Death | Stroke death   - 1 = yes - 2 = no stroke death during follow-up - 9 = insufficient data   Stroke death is coded 1 if the person died due to stroke during the follow-up.  Stroke death is coded 2 if the person did not die due to stroke during the follow-up.  Stroke death is coded 9 when there are no follow-up data available due to any reason.  Note that Stroke death= 2 includes those who survived during the follow-up or who died due to cause other than stroke. For the analysis of Stroke death as an endpoint, the date, follow-up time and age at the time of exit from the follow-up of Stroke death are the same as date of death, time to death and age at death. |  |
| Cardiovascular Death | Cardiovascular death is derived from items coronary death and stroke death as: \| \| Coronary Death \| \|; \| \| 1 \| 2 \| 9; Stroke Death \| 1 \| Cardiovascular Death = 1 \| Cardiovascular Death = 1 \| Cardiovascular Death = 1; \| 2 \| Cardiovascular Death = 1 \| Cardiovascular Death = 2 \| Cardiovascular Death = 9; \| 9 \| Cardiovascular Death = 1 \| Cardiovascular Death = 9 \| Cardiovascular Death = 9 |  |
| Cancer Death | Cancer death   - 1 = yes - 2 = no cancer death during follow-up - 9 = insufficient data   For the analysis of cancer death as an endpoint, the date, follow-up time and age at the time of exit from the follow-up of cancer death are the same as date of death, time to death and age at death. |  |

| **Table S5: Age- and sex-standardized cohorts’ baseline characteristics.** | | | | |
| --- | --- | --- | --- | --- |
|  | **Geographic regions** | | | |
|  | **Overall** | **Europe** | **Western Asia and North Africa** | **Central, East and South Asia** |
| **‍Survey year** | 1963-2019 | 1976-2015 | 1963-2015 | 1988-2019 |
| **N participants** | 798,867 | 703,470 | 42,789 | 52,608 |
| **Female sex %** | 53·7 | 53·7 | 53·7 | 53·7 |
| **Examination age (years)** | 56·8 (48·2, 63·5) | 56·7 (48·2, 63·5) | 56·8 (48·1, 63·5) | 57·0 (48·1, 64·0) |
| **Body mass index (kg/m²)** | 26·3 (23·7, 29·4) | 26·5 (23·9, 29·6) | 26·4 (23·5, 29·7) | 23·2 (21·0, 25·8) |
| **Non-HDL cholesterol (mmol/L)** | 4·2 (3·4, 4·9) | 4·2 (3·5, 5·0) | 3·9 (3·2, 47) | 3·6 (3·0, 4·3) |
| **Systolic blood pressure (mmHg)** | 134·5 (122·0, 148·0) | 135·5 (123·5, 149·5) | 122·0 (110·0, 140·0) | 125·0 (113·0, 139·0) |
| **Diastolic blood pressure (mmHg)** | 81·0 (74·5, 89·0) | 81·5 (75·0, 89·0) | 80·0 (70·0, 86·0) | 80·0 (70·0, 89·0) |
| **Current smoking %** | 17·5 | 16·8 | 15·9 | 28·0 |
| **Diabetes %** | 6·0 | 5·3 | 13·6 | 10·4 |
| **Years of education (years)** | 14·0 (10·0, 19·0) | 15·0 (10·0, 20·0) | 14·0 (12·0, 14·0) | 6·0 (0·0, 14·0) |
| **Antihypertensive medications %** | 20·3 | 20·7 | 18·9 | 16·5 |
| **Lipid-lowering medications %** | 14·3 | 15·3 | 4·7 | 8·8 |
| **History of CVD %** | 4·7 | 4·5 | 10·5 | 2·6 |
| **History of cancer %** | 8·3 | 9·4 | 0·7 | 0·1 |

Baseline characteristics were summarized using direct standardization within regions, based on the age and sex distribution of the Eurasian C&C risk collaboration dataset. Continuous variables are presented as medians with interquartile ranges (25th and 75th percentiles), except for survey year, for which the minimum and maximum values are reported. Binary variables are summarized using relative frequencies. Percentages and quartiles are calculated based on the number of available cases for each variable. Abbreviations: non-HDL, non-high-density lipoprotein; MENA, Middle East and North Africa.

| Table S6: Health examination surveys' baseline characteristics according to geographic region | | | | | |
| --- | --- | --- | --- | --- | --- |
|  |  | **Geographic regions** | | | |
|  | | **Overall** | **Europe** | **Western Asia and North Africa** | **Central, East and South Asia** |
| **N participants** | | 84,380 | 29,633 | 24,825 | 35,994 |
| **N cohorts** | | 19 | 7 | 7 | 6 |
| **Survey year, range** | | 2008-2019  NAs:0 | 2008-2018  NAs:0 | 2012-2019  NAs:0 | 2013-2024  NAs:0 |
| **Female sex %** | | 51·4  NAs:0 | 50·4  NAs:0 | 47·3  NAs:0 | 52·3  NAs:0 |
| **Examination age (years)** | | 39·0 (29·0, 51·0)  NAs:0 | 45·0 (30·0, 58·0)  NAs:0 | 32·0 (24·0, 45·0)  NAs:0 | 40·0 (30·0, 52·0)  NAs:0 |
| **Body-mass index (kg/m²)** | | 23·6 (20·5, 27·3)  NAs:2642 | 26·0 (23·1, 29·6)  NAs:748 | 24·7 (20·8, 29·4)  NAs:1153 | 22·6 (19·9, 25·6)  NAs:849 |
| **Non-HDL cholesterol (mmol/L)** | | 3·3 (2·7, 4·1)  NAs:4068 | 3·5 (2·8, 4·3)  NAs:1536 | 3·0 (2·2, 3·9)  NAs:1461 | 3·3 (2·7, 4·0)  NAs:1292 |
| **Systolic blood pressure (mmHg)** | | 121·0 (112·0, 132·0)  NAs:1475 | 123·5 (114·5, 134·0)  NAs:763 | 123·5 (115·0, 135·5)  NAs:352 | 118·5 (109·5, 129·5) NAs:437 |
| **Diastolic blood pressure (mmHg)** | | 78·0 (71·0, 85·5)  NAs:1476 | 74·5 (69·0, 82·0)  NAs:763 | 80·5 (75·0, 88·5)  NAs:352 | 78·0 (70·5, 85·5)  NAs:438 |
| **Current smoking %** | | 22·9  NAs:197 | 22·4  NAs:195 | 18·9  NAs:0 | 23·3  NAs:259 |
| **Diabetes %** | | 5·7  NAs:1612 | 6·3  NAs:93 | 7·6  NAs:1511 | 6·3  NAs:50 |
| **Years of education (years)** | | 6·0 (3·0, 10·0)  NAs:11,401 | 11·0 (10·0, 14·0)  NAs:7139 | 9·0 (5·0, 12·0)  NAs:3719 | 5·0 (2·0, 9·0)  NAs:6615 |
| **Antihypertensive medications %** | | 10·8  NAs:1943 | 19·6  NAs:466 | 8·4  NAs:704 | 10·1  NAs:815 |
| **Lipid-lowering medications %** | | 2·6  NAs:1718 | 6·5  NAs:412 | 2·8  NAs:992 | 4·6  NAs:356 |
| **History of CVD %** | | 7·5  NAs:0 | 5·8  NAs:0 | 3·4  NAs:0 | 8·2  NAs:0 |
| **History of cancer %** | | 0  NAs:0 | 0  NAs:0 | 0  NAs:0 | 1·0  NAs:0 |

Quartiles are presented for continuous variables, with the exception of survey years included where minimum and maximum are presented. Percentages are presented for binary variables. Percentages and quartiles are based on available cases per variable. Quartiles and percentages are weighted using sampling weights. To convert the values for non-HDL cholesterol from milligrams per deciliter (mg/dL) to millimoles per liter (mmol/L), multiply by 0·02586. Abbreviations: Non-HDL, non-high-density lipoprotein.

| Table S7: Cohort studies' follow-up information on cardiovascular and cancer mortality | | | | | | | | | |
| --- | --- | --- | --- | --- | --- | --- | --- | --- | --- |
| **Study** | **Survey year Range** | **AGE at examination, years Range** | **Age at end of FU, years Range** | **FU time, years Range** | **Number of individuals (with FU information) cardiovascular mortality** | **Number of cardiovascular deaths** | **Number of individuals (with FU information) cancer mortality** | **Number of cancer deaths** | **Median (25th percentile, 75th percentile) FU time, years** |
| **Overall** | 1963-2019 | 18-100 | 21-107 | 0-42·8 | 670,981 | 12,429 | 670,482 | 19674 | 8·2 (7·2, 9·6) |
| **BIRNH** | 1980-1985 | 24·9-78·6 | 27·4-88·7 | 0-10·1 | 10,798 | 246 | 10,798 | 319 | 10·1 (10·1, 10.1) |
| **CCHS 1976-1978** | 1976-1978 | 20·6-93·2 | 24·9-105·3 | 0-42·8 | 13,773 | 2488 | 13,773 | 3489 | 28·9 (16·1, 40·9) |
| **CCHS 1981-1983** | 1981-1983 | 20·8-89·4 | 26·1-101·5 | 0·3-37·7 | 1467 | 210 | 1467 | 293 | 29·5 (14·5, 36·3) |
| **CCHS 2001-2003** | 2001-2003 | 20·3-90 | 26·1-98·6 | 0·1-17·2 | 2021 | 14 | 2021 | 58 | 16·1 (15·6, 16·6) |
| **CCHS 2011-2015** | 2011-2015 | 20·8-93·7 | 23·9-98·6 | 0·1-7·9 | 1021 | 0 | 1021 | 2 | 5·3 (4·4, 6·1) |
| **ESTHER** | 2000-2003 | 48-75 | 52·1-87·6 | 0·1-12·7 | 8520 | 242 | 8520 | 395 | 11·7 (11·2, 12·2) |
| **GCS** | 2010-2012 | 42·3-87 | 44·6-93·1 | 0-9·6 | 10,013 | 288 | 10,181 | 215 | 8·0 (7·4, 8·5) |
| **HAPIEE-CZ** | 2002-2005 | 44·4-72·9 | 46-92·2 | 0·1-22·8 | 7657 | 570 | 7611 | 768 | 20·9 (19·8, 21·8) |
| **HAPIEE-Poland** | 2002-2004 | 45·1-71·6 | 46·4-82·4 | 0·1-12·9 | 8216 | 270 | 8216 | 414 | 9·3 (8·5, 10·9) |
| **Hisayama** | 1988 | 40·9-96·9 | 46·4-106·8 | 0-24 | 2572 | 174 | 2572 | 324 | 24·0 (18·2, 24·0) |
| **HUNT** | 1995-1997 | 19·1-100 | 21·3-107 | 0-23·4 | 59,582 | 3647 | 59,582 | 4081 | 22·2 (21·7, 22·9) |
| **IIHD** | 1963-1964 | 40-75 | 40·3-92·8 | 0·1-24 | 9467 | 1202 | 9467 | 645 | 23·5 (23·0, 23·7) |
| **Interepid** | 2011-2013 | 20-79·4 | 21-81·6 | 0·1-7·6 | 2165 | 27 | 2165 | 15 | 4·1 (3·2, 4·2) |
| **KHDC I and II** | 2006-2019 | 18-89 | 23·9-96·6 | 0·5-17·9 | 2733 | 67 | 2103 | 11 | 13·2 (6·2, 15·1) |
| **MDC** | 1991-1996 | 44·8-73·3 | 46·7-94 | 0-25·8 | 6473 | 135 | 6473 | 239 | 22·3 (18·1, 25·0) |
| **Moli-sani** | 2005-2010 | 34·6-98·7 | 37·3-103·2 | 0-15·8 | 22,829 | 349 | 22,828 | 830 | 13·2 (12·2, 14·2) |
| **PCS** | 2012-2015 | 37-91 | 40·1-98 | 0-7·3 | 8091 | 79 | 8091 | 45 | 5·8 (5·3, 6·1) |
| **PREVEND** | 1997-1998 | 28·8-75·7 | 30·7-93·9 | 0-19·3 | 8013 | 205 | 8013 | 586 | 18·4 (13·9, 18·8) |
| **PRIME/Lille** | 1991-1993 | 49·1-64·3 | 51·3-74·4 | 0·2-10 | 2529 | 23 | 2529 | 86 | 10·0 (10·0, 10·0) |
| **SHIP-START** | 1997-2001 | 20-81 | 24·1-99·2 | 0-25 | 4034 | 223 | 4034 | 332 | 20·4 (19·1, 22·1) |
| **SHIP-TREND** | 2008-2012 | 20-83 | 23·5-92·4 | 0-14·6 | 3842 | 52 | 3842 | 68 | 9·1 (7·6, 11·8) |
| **TLGS** | 1999-2005 | 20·1-88·9 | 22·9-100·8 | 0·2-21·1 | 10,797 | 236 | 10,797 | 147 | 19·9 (15·8, 20·5) |
| **UK Biobank** | 2006-2010 | 37·4-73·7 | 40·4-82·7 | 0-10·7 | 442,516 | 1657 | 442,516 | 6180 | 7·8 (7·1, 8·5) |
| **WHO STEPS Brunei** | 2015-2016 | 18-69 | 23·8-75·1 | 0·1-6·9 | 1857 | 2 | 1857 | 10 | 6·1 (5·9, 6·3) |
| **Yamagata** | 2010-2015 | 39-77 | 40·1-83 | 0-7 | 19,995 | 23 | 20,005 | 122 | 4·5 (3·2, 5·6) |

Quartiles of follow-up were computed using the Kaplan-Meier potential follow-up estimator. Abbreviations: FU, follow-up.

| Table S8. Risk factor-specific associations with cardiovascular and cancer mortality using individual Cox regression models by sex. | | | | | |
| --- | --- | --- | --- | --- | --- |
|  | | **Cardiovascular mortality** | | **Cancer mortality** | |
| **Exposure** | **Adjusted for** | **Women** | **Men** | **Women** | **Men** |
| **Body mass index  per 5kg/m²** | Current smoking, systolic blood pressure, antihypertensive medications, age | 0·99 (0·91, 1·07) | 1·01 (0·93, 1·08) | 1·03 (0·97, 1·10) | 1·01 (0·90, 1·12) |
| **Systolic blood pressure per 20mmHg** | Current smoking, age | 1·37 (1·22, 1·53) | 1·34 (1·25, 1·45) | 1·08 (1·06, 1·11) | 1·14 (1·06, 1·23) |
| **Non-HDL cholesterol per 1mmol/L** | Current smoking, body mass index, age | 1·08 (1·00, 1·18) | 1·08 (1·01, 1·15) | 0·99 (0·97, 1·02) | 0·95 (0·91, 0·99) |
| **Current smoking** | Age | 1·70 (1·38, 2·08) | 1·36 (0·73, 2·52) | 2·26 (1·87, 2·71) | 2·26 (1·85, 2·76) |
| **Diabetes** | Current smoking, body mass index, age | 2·52 (2·11, 2·99) | 2·36 (2·10, 2·64) | 1·21 (1·08, 1·35) | 1·35 (1·24, 1·48) |
| **Diabetes** | Current smoking, age | 2·27 (1·96, 2·63) | 2·23 (1·93, 2·57) | 1·28 (1·08, 1·52) | 1·37 (1·21, 1·55) |

Hazard ratios (95% confidence interval) are presented. Abbreviations: non-HDL-C, non-high-density lipoprotein cholesterol

| Table S9: Population-attributable fractions of modifiable risk factors for cardiovascular and cancer mortality by geographic region and sex | | | | | | | | | | | | |
| --- | --- | --- | --- | --- | --- | --- | --- | --- | --- | --- | --- | --- |
|  | **Geographic regions** | | | | | | | | | | | |
|  | **Overall** | | | **Europe** | | | **Western Asia and North Africa** | | | **Central, East and South Asia** | | |
| **Women** | | | | | | | | | | | | |
|  | **Cardiovascular** | **Cancer** | | **Cardiovascular** | **Cancer** | | **Cardiovascular** | **Cancer** | | **Cardiovascular** | **Cancer** | |
| **Joint** | 52·3 (41·9, 62·7) | 16·3 (6·0, 26·6) | | 50·7 (39·3, 62·2) | 14·7 (3·8, 25·6) | | 61·0 (51·7, 70·3) | 15·2 (5·2, 25·2) | | 52·1 (41·9, 62·3) | 17·5 (7·0, 28·0) | |
| **Body mass index  20 ≤ BMI < 25 kg/m²** | -0·7 (-9·6, 8·2) | -4·8 (-11·7, 2·1) | | -6·0 (-18·9, 6·9) | -11·6 (-21·7, -1·5) | | -3·2 (-15·9, 9·4) | -9·8 (-19·9, 0·3) | | 3·7 (-3·4, 10·7) | 0·2 (-5·9, 6·2) | |
| **Systolic blood pressure  < 120mmHg** | 42·1 (31·6, 52·6) | 7·8 (1·2, 14·4) | | 42·4 (31·5, 53·3) | 7·8 (0·9, 14·6) | | 51·1 (41·4, 60·9) | 11·3 (3·7, 19·0) | | 40·6 (30·2, 50·9) | 7·3 (1·1, 13·6) | |
| **Non-HDL cholesterol  < 3mmol/L** | -1·9 (-14·7, 10·9) | 5·1 (-5·5, 15·6) | | -0·9 (-16·8, 15·1) | 6·1 (-6·2, 18·4) | | -1·2 (-14·0, 11·5) | 4·9 (-5·2, 15·0) | | -2·8 (-13·4, 7·8) | 4·4 (-5·2, 14·0) | |
| **Current smoking** | 5·3 (3·1, 7·5) | 6·9 (5·4, 8·4) | | 7·1 (4·0, 10·1) | 10·1 (8·1, 12·2) | | 4·6 (2·6, 6·6) | 5·5 (4·1, 6·9) | | 4·1 (2·3, 5·9) | 5·1 (3·7, 6·4) | |
| **Diabetes** | 14·6 (9·7, 19·4) | 1·7 (0·0, 3·3) | | 14·0 (9·0, 18·9) | 1·6 (0·0, 3·1) | | 19·6 (13·2, 26·1) | 2·5 (0·1, 4·8) | | 14·2 (9·3, 19·1) | 1·6 (0·0, 3·3) | |
| **Men** | | | | | | | | | | | | |
|  | **Cardiovascular** | | **Cancer** | **Cardiovascular** | | **Cancer** | **Cardiovascular** | | **Cancer** | **Cardiovascular** | | **Cancer** |
| **Joint** | 44·4 (33·8, 54·9) | | 29·3 (20·0, 38·7) | 37·5 (24·1, 50·9) | | 17·2 (5·8, 28·5) | 51·2 (41·6, 60·9) | | 30·3 (20·9, 39·7) | 46·6 (36·6, 56·6) | | 34·5 (25·7, 43·3) |
| **Body mass index  20 ≤ BMI < 25 kg/m²** | 3·9 (-4·3, 12·1) | | 6·1 (0·6, 11·6) | -0·6 (-8·6, 7·4) | | 3·9 (-2·1, 9·8) | 3·2 (-4·6, 11·0) | | 6·3 (0·6, 12·0) | 6·1 (-3·4, 15·7) | | 7·0 (0·8, 13·3) |
| **Systolic blood pressure  < 120mmHg** | 16·3 (6·6, 25·9) | | 5·4 (0·1, 10·6) | 17·3 (6·5, 28·0) | | 5·9 (-0·1, 12·0) | 24·6 (13·4, 35·8) | | 8·3 (1·8, 14·7) | 14·6 (5·7, 23·5) | | 4·7 (0·0, 9·5) |
| **Non-HDL cholesterol  < 3mmol/L** | 0·7 (-9·9, 11·3) | | -6·5 (-13·4, 0·5) | 3·2 (-9·6, 16·0) | | -10·0 (-18·5, -1·5) | 1·9 (-8·1, 12·0) | | -6·3 (-12·4, -0·1) | -0·7 (-10·4, 8·9) | | -4·9 (-11·3, 1·4) |
| **Current smoking** | 20·4 (16·0, 24·7) | | 22·5 (18·6, 26·4) | 12·8 (9·6, 16·1) | | 13·9 (11·2, 16·6) | 17·5 (13·4, 21·7) | | 18·7 (14·9, 22·5) | 24·4 (19·6, 29·2) | | 26·7 (22·1, 31·3) |
| **Diabetes** | 11·2 (8·3, 14·0) | | 3·5 (1·9, 5·2) | 11·1 (8·0, 14·3) | | 3·5 (1·8, 5·2) | 16·6 (12·4, 20·9) | | 5·3 (2·9, 7·7) | 10·4 (7·5, 13·3) | | 3·3 (1·7, 5·0) |

Population-attributable fractions for 10-year mortality risks were estimated with the use of Weibull regression models. PAFs for single risk factors and aggregated PAF for the risk factors combined are shown. Abbreviations: non-HDL-C, non-high-density lipoprotein cholesterol.

| Table S10: Population-attributable fractions for cardiovascular and cancer mortality across categories of continuous modifiable risk factors | | | | | | | | | | | | |
| --- | --- | --- | --- | --- | --- | --- | --- | --- | --- | --- | --- | --- |
|  | **Geographic regions** | | | | | | | | | | | |
|  | **Overall** | | | **Europe** | | | **Western Asia and North Africa** | | | **Central, East and South Asia** | | |
| **Women** | | | | | | | | | | | | |
|  | **Cardiovascular** | **Cancer** | | **Cardiovascular** | **Cancer** | | **Cardiovascular** | **Cancer** | | **Cardiovascular** | **Cancer** | |
| **BMI < 20 kg/m^2^** | 4·8 (1·2, 8·4) | 3·9 (0·6, 7·3) | | 1·2 (-0·1, 2·4) | 1·0 (0·0, 2·0) | | 3·0 (0·6, 5·4) | 2·7 (0·3, 5·0) | | 7·8 (2·2, 13·3) | 5·9 (1·0, 10·8) | |
| **25 ≤ BMI < 30 kg/m^2^** | -4·1 (-8·6, 0·4) | -5·3 (-9·3, -1·4) | | -4·9 (-10·4, 0·6) | -6·7 (-11·8, -1·6) | | -3·7 (-7·8, 0·5) | -5·2 (-9·2, -1·2) | | -3·6 (-7·5, 0·2) | -4·5 (-7·8, -1·3) | |
| **30 kg/m² ≤ BMI** | -1·4 (-6·2, 3·4) | -3·4 (-6·5, -0·2) | | -2·3 (-10·5, 5·9) | -5·9 (-11·7, -0·2) | | -2·6 (-12·0, 6·9) | -7·2 (-14·2, -0·3) | | -0·5 (-2·1, 1·2) | -1·2 (-2·4, 0·0) | |
| **120 ≤ SBP < 140 mmHg** | 16·0 (9·2, 22·7) | 1·9 (-2·4, 6·2) | | 16·2 (9·0, 23·3) | 2·0 (-2·6, 6·6) | | 11·7 (6·4, 17·0) | 1·8 (-2·3, 5·9) | | 16·5 (9·6, 23·3) | 1·8 (-2·3, 6·0) | |
| **140 ≤ SBP < 160 mmHg** | 14·8 (10·9, 18·8) | 3·3 (1·1, 5·6) | | 17·7 (12·6, 22·8) | 3·9 (1·3, 6·4) | | 17·4 (12·6, 22·1) | 4·5 (1·5, 7·5) | | 12·3 (8·9, 15·8) | 2·8 (0·9, 4·8) | |
| **160 mmHg ≤ SBP** | 11·3 (9·2, 13·5) | 2·6 (1·3, 3·8) | | 8·6 (6·3, 10·9) | 1·9 (1·0, 2·8) | | 22·1 (18·0, 26·2) | 5·0 (2·7, 7·2) | | 11·8 (9·3, 14·3) | 2·7 (1·4, 4·0) | |
| **3 ≤ non-HDL-C < 4 mmol/L** | -3·7 (-9·3, 1·9) | 2·4 (-3·0, 7·8) | | -3·7 (-9·5, 2·1) | 2·4 (-2·9, 7·7) | | -3·6 (-9·2, 1·9) | 2·2 (-2·7, 7·2) | | -3·7 (-9·3, 1·8) | 2·5 (-3·1, 8·0) | |
| **4 ≤ non-HDL-C < 5 mmol/L** | -0·2 (-5·6, 5·3) | 1·3 (-2·5, 5·1) | | -0·1 (-7·3, 7·0) | 1·6 (-3·2, 6·4) | | -0·2 (-4·7, 4·4) | 1·1 (-2·2, 4·3) | | -0·2 (-4·5, 4·2) | 1·1 (-2·2, 4·4) | |
| **5 mmol/L ≤ non-HDL-C** | 2·0 (-1·2, 5·1) | 1·3 (-0·8, 3·5) | | 3·0 (-1·8, 7·7) | 2·0 (-1·2, 5·3) | | 2·6 (-1·5, 6·7) | 1·6 (-1·0, 4·2) | | 1·1 (-0·8, 3·0) | 0·9 (-0·6, 2·3) | |
| **Men** | | | | | | | | | | | | |
|  | **Cardiovascular** | | **Cancer** | **Cardiovascular** | | **Cancer** | **Cardiovascular** | | **Cancer** | **Cardiovascular** | | **Cancer** |
| **BMI < 20 kg/m^2^** | 5·4 (-0·4, 11·2) | | 4·8 (0·8, 8·8) | 0·5 (-0·1, 1·0) | | 0·4 (0·0, 0·8) | 3·2 (-0·4, 6·8) | | 3·0 (0·4, 5·6) | 8·0 (-0·4, 16·4) | | 7·0 (1·3, 12·7) |
| **25 ≤ BMI < 30 kg/m^2^** | -3·5 (-6·9, 0·0) | | -0·7 (-2·8, 1·4) | -5·4 (-10·7, 0·0) | | -1·2 (-4·7, 2·3) | -3·8 (-7·6, 0·0) | | -0·8 (-3·1, 1·5) | -2·5 (-5·0, 0·0) | | -0·5 (-1·9, 0·9) |
| **30 kg/m^2^ ≤ BMI** | 2·0 (0·4, 3·6) | | 2·0 (0·6, 3·4) | 4·3 (0·8, 7·8) | | 4·6 (1·4, 7·9) | 3·8 (0·7, 6·8) | | 4·1 (1·2, 7·0) | 0·6 (0·1, 1·1) | | 0·6 (0·1, 1·0) |
| **120 ≤ SBP < 140 mmHg** | 2·4 (-2·7, 7·5) | | 1·2 (-2·4, 4·7) | 2·8 (-3·2, 8·7) | | 1·4 (-2·8, 5·5) | 2·2 (-2·5, 7·0) | | 1·1 (-2·4, 4·7) | 2·2 (-2·6, 7·1) | | 1·1 (-2·2, 4·4) |
| **140 ≤ SBP < 160 mmHg** | 6·7 (3·2, 10·2) | | 2·3 (0·8, 3·8) | 8·8 (4·4, 13·2) | | 3·0 (1·1, 5·0) | 8·5 (4·1, 13·0) | | 3·2 (1·1, 5·3) | 5·4 (2·4, 8·4) | | 1·8 (0·5, 3·1) |
| **160 mmHg ≤ SBP** | 7·2 (5·1, 9·3) | | 1·9 (0·8, 3·1) | 5·7 (4·0, 7·4) | | 1·5 (0·6, 2·4) | 13·9 (10·2, 17·6) | | 3·9 (1·6, 6·2) | 6·9 (4·6, 9·2) | | 1·9 (0·7, 3·0) |
| **3 ≤ non-HDL-C < 4 mmol/L** | -2·8 (-8·2, 2·6) | | -0·1 (-4·6, 4·4) | -2·6 (-7·9, 2·6) | | -0·1 (-4·6, 4·4) | -2·4 (-7·1, 2·4) | | 0·0 (-3·9, 3·8) | -2·9 (-8·6, 2·7) | | -0·1 (-4·7, 4·5) |
| **4 ≤ non-HDL-C < 5 mmol/L** | 0·5 (-3·3, 4·3) | | -4·3 (-6·6, -2·0) | 0·8 (-4·4, 5·9) | | -6·1 (-9·5, -2·7) | 0·4 (-2·9, 3·7) | | -3·6 (-5·6, -1·7) | 0·4 (-2·9, 3·6) | | -3·6 (-5·5, -1·7) |
| **5 mmol/L ≤ non-HDL-C** | 3·0 (0·9, 5·1) | | -2·1 (-3·1, -1·1) | 5·1 (1·7, 8·5) | | -3·8 (-5·7, -2·0) | 3·9 (1·1, 6·7) | | -2·6 (-3·9, -1·3) | 1·8 (0·4, 3·3) | | -1·3 (-1·9, -0·6) |

Population-attributable fractions for 10-year mortality risks were estimated with the use of Weibull regression models. PAFs for single risk factors and aggregated PAF for the risk factors combined are shown. Abbreviations: SBP, systolic blood pressure; non-HDL-C, non-high-density lipoprotein cholesterol; BMI, body-mass index.

| Table S11: Population-attributable fractions of modifiable risk factors and education for cardiovascular and cancer mortality by geographic region and sex | | | | | | | | | | |
| --- | --- | --- | --- | --- | --- | --- | --- | --- | --- | --- |
|  | **Geographic regions** | | | | | | | | | |
|  | **Overall** | | **Europe** | | **Western Asia and North Africa** | | | **Central, East and South Asia** | | |
| **Women** | | | | | | | | | | |
|  | **Cardiovascular** | **Cancer** | **Cardiovascular** | **Cancer** | **Cardiovascular** | **Cancer** | | **Cardiovascular** | **Cancer** | |
|  | **mortality** | |  |  |  |  | |  |  | |
| **Joint** | 56·0 (43·3, 68·7) | 21·9 (9·5, 34·3) | 57·0 (45·4, 68·7) | 18·2 (6·9, 29·4) | 61·2 (50·5, 71·9) | 20·3 (9·2, 31·4) | | 54·0 (39·7, 68·2) | 22·9 (9·2, 36·6) | |
| **Body mass index  20 ≤ BMI < 25 kg/m²** | -5·1 (-19·2, 8·9) | 1·9 (-9·6, 13·4) | -14·3 (-33·0, 4·5) | -4·9 (-20·4, 10·6) | -11·8 (-28·7, 5·2) | -2·6 (-16·3, 11·2) | | -0·8 (-14·8, 13·3) | 4·0 (-8·4, 16·3) | |
| **Systolic blood pressure  < 120mmHg** | 44·0 (30·9, 57·2) | 14·2 (4·9, 23·4) | 52·9 (39·7, 66·0) | 18·2 (7·1, 29·3) | 49·5 (36·1, 62·8) | 16·8 (5·9, 27·7) | | 40·2 (26·6, 53·7) | 12·9 (4·3, 21·6) | |
| **Non-HDL cholesterol  < 3mmol/L** | 3·6 (-10·5, 17·7) | 0·0 (-11·6, 11·7) | 3·7 (-10·8, 18·3) | 0·0 (-11·6, 11·6) | 4·0 (-9·1, 17·1) | -0·5 (-11·5, 10·5) | | 3·4 (-11·0, 17·8) | 0·2 (-11·6, 12·0) | |
| **Current smoking** | 6·5 (3·3, 9·7) | 6·3 (4·0, 8·5) | 3·5 (1·6, 5·4) | 3·8 (2·4, 5·3) | 5·3 (2·5, 8·1) | 5·4 (3·5, 7·3) | | 7·6 (3·9, 11·4) | 6·8 (4·2, 9·4) | |
| **Diabetes** | 15·6 (10·3, 20·9) | 0·8 (-1·1, 2·8) | 15·1 (9·9, 20·2) | 0·8 (-1·0, 2·5) | 24·0 (16·5, 31·4) | 1·3 (-2·0, 4·7) | | 12·8 (8·0, 17·7) | 0·7 (-0·9, 2·3) | |
| **Education** | 14·4 (3·4, 25·4) | 12·9 (3·5, 22·2) | 13·9 (2·1, 25·6) | 11·9 (3·0, 20·7) | 13·8 (2·1, 25·4) | 11·7 (2·9, 20·5) | | 14·7 (4·0, 25·4) | 13·3 (3·8, 22·8) | |
| **Men** | | | | | | | | | | |
|  | **Cardiovascular** | **Cancer** | **Cardiovascular** | **Cancer** | **Cardiovascular** | | **Cancer** | **Cardiovascular** | | **Cancer** |
| **Joint** | 47·1 (32·2, 61·9) | 46·9 (32·5, 61·3) | 46·2 (32·5, 59·8) | 37·3 (21·4, 53·1) | 49·0 (35·6, 62·4) | | 34·8 (18·9, 50·6) | 46·8 (30·6, 62·9) | | 49·9 (35·8, 64·1) |
| **Body mass index  20 ≤ BMI < 25 kg/m²** | 3·4 (-13·8, 20·6) | 12·8 (1·5, 24·1) | -1·6 (-13·3, 10·1) | -0·4 (-9·2, 8·3) | 0·0 (-12·3, 12·3) | | 3·9 (-4·8, 12·7) | 4·8 (-15·4, 25·0) | | 15·7 (3·1, 28·3) |
| **Systolic blood pressure  < 120mmHg** | 18·3 (3·1, 33·6) | 8·6 (-1·2, 18·4) | 26·7 (8·7, 44·7) | 12·4 (-0·8, 25·6) | 24·2 (6·4, 42·0) | | 11·3 (-1·5, 24·1) | 15·9 (1·5, 30·3) | | 7·7 (-1·2, 16·7) |
| **Non-HDL cholesterol  < 3mmol/L** | -4·5 (-14·0, 4·9) | 2·1 (-10·2, 14·3) | -4·0 (-13·5, 5·5) | 1·7 (-10·7, 14·0) | -2·0 (-11·1, 7·2) | | 1·2 (-10·1, 12·5) | -5·2 (-14·7, 4·3) | | 2·3 (-10·2, 14·7) |
| **Current smoking** | 27·6 (21·9, 33·2) | 30·7 (24·0, 37·4) | 23·8 (18·8, 28·9) | 26·6 (20·5, 32·7) | 18·6 (13·8, 23·4) | | 19·5 (14·6, 24·4) | 30·1 (24·1, 36·1) | | 33·0 (25·9, 40·1) |
| **Diabetes** | 9·7 (6·4, 13·0) | 1·4 (0·2, 2·6) | 8·7 (5·4, 11·9) | 1·2 (0·2, 2·1) | 18·7 (12·8, 24·6) | | 2·9 (0·5, 5·3) | 7·8 (4·6, 10·9) | | 1·2 (0·1, 2·2) |
| **Education** | 13·0 (5·4, 20·5) | 5·8 (-3·3, 15·0) | 13·2 (5·8, 20·6) | 6·0 (-2·9, 14·8) | 12·1 (5·0, 19·1) | | 5·0 (-3·5, 13·6) | 13·1 (5·4, 20·8) | | 5·9 (-3·4, 15·2) |

Population-attributable fractions for 10-year mortality risks were estimated with the use of Weibull regression models. PAFs for single risk factors and aggregated PAF for the risk factors combined are shown. Abbreviations: non-HDL-C, non-high-density lipoprotein cholesterol.

| Table S12: Population-attributable fractions across categories of continuous modifiable risk factors and education for cardiovascular and cancer mortality | | | | | | | | | | | | |
| --- | --- | --- | --- | --- | --- | --- | --- | --- | --- | --- | --- | --- |
|  | **Geographic regions** | | | | | | | | | | | |
|  | **Overall** | | | **Europe** | | | **Western Asia and North Africa** | | | **Central, East and South Asia** | | |
| **Women** | | | | | | | | | | | | |
|  | **Cardiovascular** | **Cancer** | | **Cardiovascular** | **Cancer** | | **Cardiovascular** | **Cancer** | | **Cardiovascular** | **Cancer** | |
| **BMI < 20 kg/m^2^** | 4·8 (-3·2, 12·8) | 5·6 (-3·6, 14·8) | | 0·5 (-0·4, 1·5) | 0·9 (-0·8, 2·6) | | 1·5 (-1·3, 4·4) | 2·3 (-1·8, 6·4) | | 6·9 (-4·0, 17·9) | 7·1 (-4·2, 18·4) | |
| **25 ≤ BMI < 30 kg/m^2^** | -6·8 (-12·3, -1·3) | -3·1 (-7·9, 1·7) | | -8·2 (-15·2, -1·2) | -4·3 (-11·3, 2·7) | | -7·3 (-13·3, -1·2) | -3·4 (-8·9, 2·1) | | -6·4 (-11·5, -1·2) | -2·8 (-7·2, 1·5) | |
| **30 kg/m^2^ ≤ BMI** | -3·1 (-8·9, 2·7) | -0·6 (-4·4, 3·1) | | -6·6 (-19·2, 6·0) | -1·5 (-10·6, 7·5) | | -6·0 (-17·4, 5·4) | -1·5 (-10·4, 7·4) | | -1·4 (-4·0, 1·3) | -0·3 (-2·1, 1·5) | |
| **120 ≤ SBP < 140 mmHg** | 9·2 (2·5, 15·9) | 6·1 (0·9, 11·3) | | 7·6 (2·0, 13·1) | 6·4 (1·0, 11·8) | | 8·4 (2·1, 14·7) | 6·4 (0·8, 12·0) | | 9·8 (2·7, 16·9) | 5·9 (0·8, 11·1) | |
| **140 ≤ SBP < 160 mmHg** | 13·9 (8·9, 18·8) | 4·1 (0·9, 7·2) | | 15·8 (10·5, 21·0) | 5·4 (1·3, 9·5) | | 16·9 (11·0, 22·8) | 5·4 (1·3, 9·5) | | 12·4 (7·4, 17·3) | 3·5 (0·7, 6·4) | |
| **160 mmHg ≤ SBP** | 21·0 (15·3, 26·7) | 4·1 (2·1, 6·1) | | 29·5 (22·8, 36·2) | 6·4 (3·4, 9·5) | | 24·2 (18·0, 30·4) | 5·0 (2·5, 7·5) | | 18·0 (11·8, 24·1) | 3·5 (1·7, 5·3) | |
| **3 ≤ non-HDL-C < 4 mmol/L** | 0·1 (-8·1, 8·4) | 2·9 (-3·2, 9·0) | | 0·1 (-8·3, 8·6) | 2·9 (-3·2, 8·9) | | 0·2 (-7·3, 7·7) | 2·6 (-2·8, 7·9) | | 0·1 (-8·3, 8·6) | 3·0 (-3·3, 9·3) | |
| **4 ≤ non-HDL-C < 5 mmol/L** | 1·6 (-3·6, 6·8) | -1·8 (-6·1, 2·6) | | 1·7 (-3·8, 7·1) | -1·8 (-6·1, 2·6) | | 1·3 (-2·9, 5·6) | -1·6 (-5·4, 2·3) | | 1·6 (-3·9, 7·1) | -1·8 (-6·2, 2·6) | |
| **5 mmol/L ≤ non-HDL-C** | 1·9 (-0·9, 4·6) | -1·1 (-3·0, 0·8) | | 1·9 (-0·9, 4·7) | -1·1 (-3·0, 0·8) | | 2·5 (-1·0, 6·0) | -1·5 (-4·1, 1·1) | | 1·6 (-0·9, 4·2) | -1·0 (-2·8, 0·7) | |
| **Thirds of years of education: lowest category** | 5·5 (-2·2, 13·2) | 4·8 (-0·6, 10·2) | | 6·8 (-2·5, 16·2) | 5·0 (-0·5, 10·5) | | 6·2 (-2·6, 14·9) | 5·0 (-0·6, 10·6) | | 5·0 (-2·0, 11·9) | 4·7 (-0·6, 10·1) | |
| **Thirds of years of education: middle category** | 8·9 (3·7, 14·1) | 8·1 (2·4, 13·7) | | 7·0 (2·8, 11·2) | 6·9 (1·9, 11·8) | | 7·6 (2·7, 12·5) | 6·7 (1·8, 11·5) | | 9·8 (4·1, 15·4) | 8·6 (2·6, 14·6) | |
| **Men** | | | | | | | | | | | | |
|  | **Cardiovascular** | | **Cancer** | **Cardiovascular** | | **Cancer** | **Cardiovascular** | | **Cancer** | **Cardiovascular** | | **Cancer** |
| **BMI < 20 kg/m^2^** | 4·6 (-11·1, 20·4) | | 13·5 (2·9, 24·1) | 0·5 (-1·5, 2·6) | | 1·6 (0·0, 3·2) | 1·5 (-4·5, 7·6) | | 5·5 (0·6, 10·5) | 5·9 (-13·6, 25·4) | | 16·1 (3·8, 28·3) |
| **25 ≤ BMI < 30 kg/m^2^** | -1·7 (-5·4, 2·0) | | -0·5 (-2·2, 1·3) | -3·4 (-10·5, 3·8) | | -1·2 (-5·7, 3·2) | -2·7 (-8·7, 3·3) | | -0·8 (-4·2, 2·5) | -1·2 (-3·9, 1·5) | | -0·3 (-1·5, 0·9) |
| **30 kg/m^2^ ≤ BMI** | 0·4 (-1·2, 2·1) | | -0·2 (-1·5, 1·1) | 1·2 (-3·6, 6·0) | | -0·8 (-5·7, 4·1) | 1·2 (-3·3, 5·7) | | -0·8 (-5·3, 3·8) | 0·1 (-0·4, 0·7) | | -0·1 (-0·5, 0·3) |
| **120 ≤ SBP < 140 mmHg** | 2·5 (-5·3, 10·2) | | 3·8 (-1·8, 9·4) | 2·4 (-5·3, 10·1) | | 2·4 (-5·3, 10·1) | 2·7 (-5·7, 11·0) | | 4·4 (-2·1, 10·9) | 2·4 (-5·3, 10·1) | | 3·6 (-1·8, 9·1) |
| **140 ≤ SBP < 160 mmHg** | 5·9 (0·6, 11·2) | | 2·2 (-0·4, 4·8) | 8·4 (1·4, 15·4) | | 8·4 (1·4, 15·4) | 7·8 (1·1, 14·5) | | 3·2 (-0·6, 7·0) | 5·1 (0·3, 9·9) | | 1·9 (-0·4, 4·2) |
| **160 mmHg ≤ SBP** | 10·0 (6·0, 14·0) | | 2·6 (-0·1, 5·2) | 15·8 (10·5, 21·1) | | 15·8 (10·5, 21·1) | 13·7 (8·8, 18·6) | | 3·7 (-0·3, 7·7) | 8·4 (4·6, 12·1) | | 2·2 (-0·1, 4·5) |
| **3 ≤ non-HDL-C < 4 mmol/L** | -4·4 (-10·3, 1·6) | | 3·2 (-5·7, 12·0) | -4·1 (-9·6, 1·3) | | 3·0 (-5·5, 11·5) | -3·3 (-8·1, 1·5) | | 2·7 (-4·3, 9·7) | -4·6 (-10·9, 1·6) | | 3·3 (-5·9, 12·5) |
| **4 ≤ non-HDL-C < 5 mmol/L** | -1·8 (-5·1, 1·4) | | -0·5 (-3·5, 2·4) | -2·0 (-5·4, 1·5) | | -0·6 (-3·8, 2·6) | -1·7 (-4·8, 1·4) | | -0·5 (-3·6, 2·6) | -1·8 (-5·1, 1·4) | | -0·6 (-3·5, 2·4) |
| **5 mmol/L ≤ non-HDL-C** | 1·6 (0·1, 3·2) | | -0·6 (-1·6, 0·5) | 2·1 (0·2, 4·0) | | -0·7 (-2·0, 0·6) | 3·0 (0·3, 5·7) | | -1·0 (-3·0, 1·0) | 1·3 (0·0, 2·5) | | -0·5 (-1·3, 0·4) |
| **Thirds of years of education: lowest category** | 8·5 (2·4, 14·6) | | 5·6 (0·0, 11·1) | 9·1 (2·9, 15·2) | | 5·6 (0·0, 11·3) | 7·5 (2·0, 13·0) | | 4·7 (-0·3, 9·8) | 8·7 (2·4, 14·9) | | 5·7 (0·0, 11·4) |
| **Thirds of years of education: middle category** | 4·4 (1·0, 7·9) | | 0·3 (-3·9, 4·4) | 4·1 (1·0, 7·2) | | 0·3 (-3·4, 4·0) | 4·6 (1·1, 8·0) | | 0·3 (-3·7, 4·4) | 4·5 (0·9, 8·0) | | 0·2 (-4·0, 4·5) |

Population-attributable fractions for 10-year mortality risks were estimated with the use of Weibull regression models. PAFs for single risk factors and aggregated PAF for the risk factors combined are shown. Abbreviations: SBP, systolic blood pressure; non-HDL-C, non-high-density lipoprotein cholesterol; BMI, body-mass index.

| Table S13: Population-attributable fractions for premature cardiovascular and cancer mortality by geographic region and sex | | | | | | | | | | | | |
| --- | --- | --- | --- | --- | --- | --- | --- | --- | --- | --- | --- | --- |
|  | **Geographic regions** | | | | | | | | | | | |
|  | **Overall** | | | **Europe** | | | **Western Asia and North Africa** | | | **Central, East and South Asia** | | |
| **Women** | | | | | | | | | | | | |
|  | **Cardiovascular** | **Cancer** | | **Cardiovascular** | **Cancer** | | **Cardiovascular** | **Cancer** | | **Cardiovascular** | **Cancer** | |
| **Joint** | 49·9 (38·2, 61·7) | 17·5 (6·4, 28·6) | | 39·6 (24·9, 54·4) | 14·0 (1·8, 26·2) | | 59·9 (49·4, 70·3) | 17·0 (5·7, 28·3) | | 50·2 (38·0, 62·4) | 18·5 (7·0, 30·1) | |
| **Body mass index  20 ≤ BMI < 25 kg/m²** | 1·8 (-7·5, 11·0) | -3·2 (-10·4, 4·0) | | -4·9 (-16·3, 6·4) | -10·4 (-19·9, -1·0) | | -1·1 (-13·1, 10·9) | -7·4 (-17·1, 2·3) | | 4·0 (-5·1, 13·1) | -0·4 (-7·6, 6·8) | |
| **Systolic blood pressure  < 120mmHg** | 40·9 (30·9, 51·0) | 8·0 (1·8, 14·2) | | 36·0 (24·2, 47·8) | 5·7 (-1·1, 12·5) | | 51·3 (41·1, 61·6) | 13·0 (4·8, 21·1) | | 39·8 (29·6, 49·9) | 7·8 (1·9, 13·6) | |
| **Non-HDL cholesterol  < 3mmol/L** | -3·9 (-18·4, 10·6) | 5·3 (-6·2, 16·7) | | -4·7 (-20·2, 10·7) | 5·4 (-6·7, 17·5) | | -2·3 (-16·0, 11·4) | 5·0 (-5·5, 15·6) | | -4·0 (-18·6, 10·5) | 5·3 (-6·2, 16·7) | |
| **Current smoking** | 6·1 (2·7, 9·5) | 6·4 (4·2, 8·6) | | 10·2 (4·3, 16·2) | 12·2 (6·5, 18·0) | | 4·6 (0·0, 9·2) | 3·7 (1·0, 6·3) | | 5·4 (1·6, 9·2) | 5·2 (2·9, 7·4) | |
| **Diabetes** | 11·7 (6·2, 17·2) | 1·5 (-0·1, 3·0) | | 3·5 (-0·1, 7·1) | 0·4 (-0·2, 0·9) | | 16·0 (8·3, 23·7) | 2·3 (0·0, 4·6) | | 12·7 (5·8, 19·6) | 1·6 (-0·2, 3·4) | |
| **Men** | | | | | | | | | | | | |
|  | **Cardiovascular** | | **Cancer** | **Cardiovascular** | | **Cancer** | **Cardiovascular** | | **Cancer** | **Cardiovascular** | | **Cancer** |
| **Joint** | 42·7 (31·9, 53·5) | | 31·7 (22·1, 41·4) | 38·4 (21·2, 55·6) | | 25·4 (10·4, 40·4) | 43·9 (32·2, 55·5) | | 25·6 (15·6, 35·7) | 44·1 (33·8, 54·4) | | 34·8 (25·7, 44·0) |
| **Body mass index  20 ≤ BMI < 25 kg/m²** | 4·7 (-3·5, 12·8) | | 5·8 (0·4, 11·2) | 2·8 (-7·3, 13·0) | | 4·9 (-1·4, 11·2) | 6·2 (-2·5, 14·9) | | 7·9 (1·6, 14·1) | 5·1 (-3·3, 13·5) | | 5·9 (0·3, 11·5) |
| **Systolic blood pressure  < 120mmHg** | 15·5 (5·8, 25·2) | | 4·9 (0·0, 9·9) | 19·0 (4·9, 33·0) | | 6·2 (-0·9, 13·3) | 16·9 (5·5, 28·4) | | 5·8 (-0·1, 11·6) | 14·0 (5·1, 22·9) | | 4·4 (0·1, 8·6) |
| **Non-HDL cholesterol  < 3mmol/L** | -0·4 (-11·3, 10·5) | | -6·0 (-13·2, 1·1) | 0·5 (-12·5, 13·6) | | -6·8 (-15·5, 2·0) | 1·9 (-7·2, 11·0) | | -6·3 (-11·9, -0·7) | -1·1 (-11·7, 9·5) | | -5·7 (-12·8, 1·3) |
| **Current smoking** | 23·7 (17·9, 29·5) | | 26·6 (20·7, 32·4) | 19·9 (11·9, 27·9) | | 21·5 (13·6, 29·4) | 13·2 (7·9, 18·5) | | 14·7 (8·8, 20·5) | 26·8 (20·0, 33·5) | | 30·0 (23·4, 36·7) |
| **Diabetes** | 5·6 (2·7, 8·5) | | 1·7 (0·5, 2·9) | 4·1 (0·5, 7·8) | | 1·1 (-0·1, 2·4) | 14·5 (7·2, 21·8) | | 4·6 (1·8, 7·5) | 4·8 (1·1, 8·5) | | 1·5 (0·1, 2·9) |

Population-attributable fractions for 20-year mortality risks at index age 50 were estimated with the use of Weibull regression models. PAFs for single risk factors and aggregated PAF for the risk factors combined are shown. Abbreviations: non-HDL-C, non-high-density lipoprotein cholesterol.

| Table S14: Population-attributable fractions across categories of continuous classic modifiable risk factors for premature cardiovascular and cancer mortality | | | | | | | | | | | | |
| --- | --- | --- | --- | --- | --- | --- | --- | --- | --- | --- | --- | --- |
|  | **Geographic regions** | | | | | | | | | | | |
|  | **Overall** | | | **Europe** | | | **Western Asia and North Africa** | | | **Central, East and South Asia** | | |
| **Women** | | | | | | | | | | | | |
|  | **Cardiovascular** | **Cancer** | | **Cardiovascular** | **Cancer** | | **Cardiovascular** | **Cancer** | | **Cardiovascular** | **Cancer** | |
| **BMI < 20 kg/m^2^** | 6·8 (1·8, 11·8) | 4·7 (0·5, 8·9) | | 1·1 (-0·6, 2·9) | 0·6 (-0·3, 1·5) | | 4·9 (1·1, 8·8) | 3·9 (0·3, 7·4) | | 8·6 (2·2, 15·0) | 6·0 (0·7, 11·3) | |
| **25 ≤ BMI < 30 kg/m^2^** | -3·8 (-8·1, 0·5) | -4·7 (-8·5, -1·0) | | -4·1 (-8·9, 0·8) | -5·7 (-10·4, -0·9) | | -3·9 (-8·5, 0·7) | -5·2 (-9·4, -0·9) | | -3·7 (-8·1, 0·6) | -4·4 (-8·1, -0·7) | |
| **30 kg/m^2^ ≤ BMI** | -1·2 (-5·8, 3·4) | -3·1 (-6·2, -0·1) | | -2·0 (-9·6, 5·6) | -5·4 (-11·1, 0·3) | | -2·1 (-10·2, 5·9) | -6·1 (-12·2, -0·1) | | -0·9 (-4·1, 2·4) | -2·0 (-4·1, 0·1) | |
| **120 ≤ SBP < 140 mmHg** | 13·4 (7·0, 19·9) | 1·6 (-2·1, 5·4) | | 21·1 (10·7, 31·5) | 2·4 (-3·3, 8·0) | | 12·5 (6·1, 18·9) | 1·8 (-2·2, 5·7) | | 11·8 (5·6, 17·9) | 1·4 (-1·8, 4·6) | |
| **140 ≤ SBP < 160 mmHg** | 12·5 (7·7, 17·3) | 3·4 (0·9, 5·8) | | 10·1 (5·1, 15·2) | 2·4 (0·5, 4·3) | | 16·1 (9·7, 22·4) | 5·1 (1·3, 8·8) | | 12·2 (6·5, 18·0) | 3·3 (0·8, 5·9) | |
| **160 mmHg ≤ SBP** | 15·0 (9·9, 20·0) | 3·0 (1·3, 4·7) | | 4·7 (1·6, 7·8) | 0·9 (0·2, 1·6) | | 22·8 (15·0, 30·6) | 6·1 (2·9, 9·4) | | 15·7 (8·9, 22·6) | 3·0 (1·1, 5·0) | |
| **3 ≤ non-HDL-C < 4 mmol/L** | -5·0 (-12·5, 2·6) | 3·0 (-3·8, 9·8) | | -5·2 (-13·5, 3·0) | 3·1 (-4·0, 10·3) | | -4·1 (-10·5, 2·2) | 2·4 (-3·0, 7·8) | | -5·1 (-12·9, 2·7) | 3·0 (-4·0, 10·1) | |
| **4 ≤ non-HDL-C < 5 mmol/L** | -0·3 (-6·7, 6·2) | 1·3 (-2·6, 5·2) | | -0·3 (-7·9, 7·3) | 1·6 (-3·3, 6·4) | | -0·2 (-5·7, 5·3) | 1·2 (-2·4, 4·9) | | -0·3 (-6·7, 6·2) | 1·2 (-2·5, 4·9) | |
| **5 mmol/L ≤ non-HDL-C** | 1·4 (-1·0, 3·7) | 1·0 (-0·7, 2·7) | | 0·9 (-0·8, 2·5) | 0·7 (-0·7, 2·1) | | 2·0 (-1·7, 5·8) | 1·4 (-1·0, 3·9) | | 1·3 (-1·0, 3·7) | 1·0 (-0·7, 2·7) | |
| Men | | | | | | | | | | | | |
|  | **Cardiovascular** | | **Cancer** | **Cardiovascular** | | **Cancer** | **Cardiovascular** | | **Cancer** | **Cardiovascular** | | **Cancer** |
| **BMI < 20 kg/m^2^** | 5·8 (-0·6, 12·3) | | 4·8 (0·6, 9·0) | 4·1 (-2·5, 10·7) | | 2·7 (-1·4, 6·9) | 5·0 (-0·6, 10·7) | | 4·6 (0·5, 8·6) | 6·6 (-0·8, 14·0) | | 5·6 (0·7, 10·5) |
| **25 ≤ BMI < 30 kg/m^2^** | -2·8 (-5·5, -0·1) | | -0·6 (-2·4, 1·2) | -4·4 (-9·2, 0·3) | | -1·1 (-4·2, 2·1) | -2·5 (-5·2, 0·2) | | -0·5 (-2·0, 1·0) | -2·2 (-4·4, 0·0) | | -0·5 (-1·8, 0·9) |
| **30 kg/m^2^ ≤ BMI** | 1·6 (0·2, 3·1) | | 1·6 (0·3, 2·9) | 3·2 (0·2, 6·1) | | 3·2 (0·7, 5·7) | 3·7 (0·4, 7·0) | | 3·8 (0·7, 6·8) | 0·7 (-0·2, 1·6) | | 0·7 (-0·2, 1·6) |
| **120 ≤ SBP < 140 mmHg** | 2·2 (-2·4, 6·7) | | 1·0 (-2·2, 4·2) | 2·9 (-3·6, 9·4) | | 1·5 (-3·2, 6·2) | 2·7 (-3·0, 8·3) | | 1·3 (-2·6, 5·2) | 1·8 (-1·9, 5·6) | | 0·9 (-1·7, 3·4) |
| **140 ≤ SBP < 160 mmHg** | 4·9 (2·0, 7·9) | | 1·7 (0·5, 2·9) | 6·2 (1·8, 10·7) | | 2·1 (0·4, 3·8) | 9·2 (3·2, 15·2) | | 3·1 (0·8, 5·4) | 3·8 (1·2, 6·4) | | 1·3 (0·2, 2·4) |
| **160 mmHg ≤ SBP** | 8·4 (3·7, 13·0) | | 2·2 (0·4, 4·0) | 9·8 (-0·8, 20·4) | | 2·7 (-1·0, 6·4) | 5·1 (1·4, 8·7) | | 1·3 (0·2, 2·4) | 8·4 (3·2, 13·6) | | 2·2 (0·4, 4·0) |
| **3 ≤ non-HDL-C < 4 mmol/L** | -3·1 (-9·0, 2·9) | | -0·1 (-4·8, 4·6) | -3·7 (-11·2, 3·8) | | -0·1 (-5·9, 5·8) | -1·7 (-5·0, 1·6) | | -0·1 (-2·8, 2·7) | -3·0 (-8·9, 2·9) | | -0·1 (-4·7, 4·5) |
| **4 ≤ non-HDL-C < 5 mmol/L** | 0·6 (-3·7, 4·8) | | -4·6 (-7·2, -1·9) | 0·4 (-3·1, 3·8) | | -4·0 (-6·7, -1·3) | 0·6 (-3·6, 4·8) | | -4·5 (-7·5, -1·5) | 0·6 (-4·0, 5·3) | | -4·8 (-7·7, -1·8) |
| **5 mmol/L ≤ non-HDL-C** | 2·1 (0·5, 3·7) | | -1·4 (-2·3, -0·6) | 3·9 (0·2, 7·6) | | -2·7 (-4·9, -0·6) | 3·0 (-0·1, 6·1) | | -1·7 (-3·2, -0·3) | 1·3 (0·2, 2·4) | | -0·9 (-1·6, -0·3) |

Population-attributable fractions for 20-year mortality risks at age 50 were estimated with the use of Weibull regression models. PAFs for single risk factors and aggregated PAF for the risk factors combined are shown. Abbreviations: SBP, systolic blood pressure; non-HDL-C, non-high-density lipoprotein cholesterol; BMI, body-mass index.

| Table S15: Population-attributable fractions of classic modifiable risk factors and education for premature cardiovascular and cancer mortality by geographic region and sex | | | | | | | | | | | | |
| --- | --- | --- | --- | --- | --- | --- | --- | --- | --- | --- | --- | --- |
|  | **Geographic regions** | | | | | | | | | | | |
|  | **Overall** | | | **Europe** | | | **Western Asia and North Africa** | | | **Central, East and South Asia** | | |
| **Women** | | | | | | | | | | | | |
|  | **Cardiovascular** | **Cancer** | | **Cardiovascular** | **Cancer** | | **Cardiovascular** | **Cancer** | | **Cardiovascular** | **Cancer** | |
| **Joint** | 52·0 (36·5, 67·6) | 20·7 (7·0, 34·3) | | 41·2 (24·1, 58·3) | 15·1 (3·3, 26·9) | | 58·9 (45·1, 72·7) | 21·0 (8·0, 33·9) | | 51·0 (34·5, 67·6) | 21·0 (6·7, 35·3) | |
| **Body mass index  20 ≤ BMI < 25 kg/m²** | -6·1 (-21·6, 9·4) | 1·5 (-9·1, 12·0) | | -15·5 (-36·2, 5·2) | -6·1 (-22·0, 9·8) | | -10·6 (-28·6, 7·4) | -1·0 (-13·1, 11·1) | | -4·4 (-19·7, 10·8) | 2·5 (-8·3, 13·3) | |
| **Systolic blood pressure  < 120mmHg** | 41·1 (26·8, 55·4) | 13·9 (4·5, 23·3) | | 43·3 (27·7, 58·9) | 16·8 (5·2, 28·4) | | 49·1 (34·7, 63·6) | 18·0 (6·6, 29·4) | | 39·0 (24·3, 53·6) | 12·9 (3·9, 21·8) | |
| **Non-HDL cholesterol  < 3mmol/L** | 3·3 (-12·4, 19·1) | 0·7 (-11·8, 13·2) | | 2·1 (-11·6, 15·7) | 1·0 (-10·2, 12·1) | | 3·8 (-10·6, 18·1) | -0·4 (-12·0, 11·3) | | 3·3 (-13·0, 19·6) | 0·9 (-12·0, 13·7) | |
| **Current smoking** | 5·1 (1·8, 8·3) | 4·5 (2·1, 6·8) | | 2·6 (0·1, 5·1) | 2·4 (0·3, 4·6) | | 5·5 (-0·5, 11·6) | 3·2 (0·5, 5·9) | | 5·1 (1·6, 8·6) | 4·9 (2·2, 7·6) | |
| **Diabetes** | 15·3 (8·2, 22·5) | 0·9 (-1·4, 3·2) | | 5·6 (1·0, 10·1) | 0·3 (-0·5, 1·1) | | 19·6 (10·4, 28·7) | 1·2 (-1·7, 4·2) | | 14·9 (6·7, 23·1) | 0·9 (-1·4, 3·2) | |
| **Education** | 14·3 (-1·0, 29·5) | 12·1 (2·0, 22·2) | | 13·1 (2·7, 23·5) | 11·4 (3·3, 19·4) | | 13·1 (0·7, 25·5) | 11·2 (2·6, 19·8) | | 14·7 (-1·7, 31·0) | 12·3 (1·7, 22·9) | |
| Men | | | | | | | | | | | | |
|  | **Cardiovascular** | | **Cancer** | **Cardiovascular** | | **Cancer** | **Cardiovascular** | | **Cancer** | **Cardiovascular** | | **Cancer** |
| **Joint** | 41·6 (27·1, 56·2) | | 42·7 (28·4, 57·1) | 43·0 (28·0, 58·1) | | 36·5 (20·6, 52·4) | 39·3 (22·4, 56·2) | | 31·8 (15·9, 47·6) | 41·9 (27·0, 56·8) | | 44·9 (30·5, 59·2) |
| **Body mass index  20 ≤ BMI < 25 kg/m²** | 2·3 (-11·6, 16·3) | | 9·7 (0·0, 19·4) | -0·7 (-10·7, 9·3) | | 0·6 (-7·2, 8·5) | 2·0 (-11·5, 15·6) | | 7·2 (-2·9, 17·3) | 2·7 (-12·3, 17·6) | | 10·8 (0·5, 21·2) |
| **Systolic blood pressure  < 120mmHg** | 15·5 (1·0, 30·0) | | 7·4 (-1·7, 16·4) | 22·4 (3·7, 41·1) | | 11·5 (-1·9, 24·8) | 16·4 (-1·5, 34·4) | | 9·4 (-2·0, 20·7) | 14·6 (0·8, 28·5) | | 6·7 (-1·7, 15·1) |
| **Non-HDL cholesterol  < 3mmol/L** | -4·9 (-14·6, 4·8) | | 1·7 (-10·1, 13·5) | -3·1 (-12·0, 5·7) | | 1·9 (-9·2, 13·0) | -2·5 (-11·1, 6·1) | | 0·5 (-9·0, 10·0) | -5·5 (-15·6, 4·6) | | 1·9 (-10·4, 14·1) |
| **Current smoking** | 25·8 (18·6, 33·0) | | 28·9 (20·7, 37·2) | 23·4 (16·0, 30·8) | | 25·1 (17·2, 33·0) | 13·8 (7·3, 20·4) | | 15·5 (8·7, 22·4) | 28·1 (20·1, 36·1) | | 31·3 (22·4, 40·1) |
| **Diabetes** | 7·1 (2·5, 11·7) | | 1·0 (0·0, 2·0) | 8·8 (1·7, 15·8) | | 1·1 (-0·3, 2·5) | 15·5 (7·6, 23·3) | | 2·5 (0·3, 4·7) | 5·5 (0·3, 10·7) | | 0·8 (-0·2, 1·8) |
| **Education** | 14·4 (5·4, 23·5) | | 7·4 (-3·1, 17·9) | 13·4 (5·1, 21·7) | | 6·4 (-2·6, 15·4) | 11·4 (4·2, 18·7) | | 5·0 (-4·1, 14·1) | 15·0 (5·5, 24·6) | | 7·8 (-3·0, 18·7) |

Population-attributable fractions for 20-year mortality risks at age 50 were estimated with the use of Weibull regression models. PAFs for single risk factors and aggregated PAF for the risk factors combined are shown. Abbreviations: non-HDL-C, non-high-density lipoprotein cholesterol.

| Table S16: Population-attributable fractions across categories of continuous modifiable risk factors and education for premature cardiovascular and cancer mortality | | | | | | | | | | | | |  |
| --- | --- | --- | --- | --- | --- | --- | --- | --- | --- | --- | --- | --- | --- |
|  | **Geographic regions** | | | | | | | | | | | |  |
|  | **Overall** | | | **Europe** | | | **Western Asia and North Africa** | | | **Central, East and South Asia** | | |  |
| **Women** | | | | | | | | | | | | |  |
|  | **Cardiovascular** | **Cancer** | | **Cardiovascular** | **Cancer** | | **Cardiovascular** | **Cancer** | | **Cardiovascular** | **Cancer** | |  |
| **BMI < 20 kg/m^2^** | 4·5 (-3·3, 12·3) | 5·1 (-3·4, 13·6) | | 0·2 (-0·4, 0·8) | 0·2 (-0·3, 0·7) | | 2·6 (-2·1, 7·3) | 3·6 (-2·8, 10·0) | | 5·2 (-3·8, 14·2) | 5·8 (-3·7, 15·2) | |  |
| **25 ≤ BMI < 30 kg/m^2^** | -8·1 (-15·8, -0·3) | -3·1 (-7·6, 1·5) | | -10·0 (-20·0, 0·0) | -4·8 (-12·6, 2·9) | | -8·1 (-16·0, -0·2) | -3·4 (-8·6, 1·9) | | -7·9 (-15·8, 0·0) | -2·9 (-7·2, 1·4) | |  |
| **30 kg/m^2^ ≤ BMI** | -2·5 (-7·8, 2·8) | -0·6 (-3·9, 2·7) | | -5·7 (-17·8, 6·3) | -1·5 (-10·3, 7·3) | | -5·1 (-15·5, 5·3) | -1·2 (-8·2, 5·7) | | -1·7 (-5·4, 2·1) | -0·4 (-2·7, 1·9) | |  |
| **120 ≤ SBP < 140 mmHg** | 9·7 (1·7, 17·6) | 5·7 (0·6, 10·7) | | 13·9 (3·6, 24·2) | 8·7 (1·4, 15·9) | | 9·3 (1·9, 16·7) | 6·3 (1·0, 11·7) | | 9·5 (1·3, 17·7) | 5·3 (0·4, 10·3) | |  |
| **140 ≤ SBP < 160 mmHg** | 13·6 (7·4, 19·8) | 4·8 (1·0, 8·5) | | 15·2 (7·8, 22·7) | 5·1 (1·0, 9·1) | | 15·5 (8·6, 22·5) | 5·9 (1·1, 10·7) | | 13·0 (6·2, 19·9) | 4·5 (0·8, 8·2) | |  |
| **160 mmHg ≤ SBP** | 17·8 (9·8, 25·8) | 3·5 (1·3, 5·7) | | 14·2 (6·7, 21·6) | 3·1 (0·9, 5·3) | | 24·3 (14·0, 34·6) | 5·8 (2·1, 9·5) | | 16·4 (7·4, 25·5) | 3·0 (0·9, 5·2) | |  |
| **3 ≤ non-HDL-C < 4 mmol/L** | 0·2 (-9·3, 9·6) | 3·4 (-3·8, 10·6) | | 0·2 (-8·9, 9·2) | 3·1 (-3·7, 9·9) | | 0·1 (-7·8, 8·1) | 2·7 (-3·1, 8·5) | | 0·2 (-9·7, 10·1) | 3·6 (-4·0, 11·1) | |  |
| **4 ≤ non-HDL-C < 5 mmol/L** | 1·9 (-4·5, 8·4) | -1·8 (-6·3, 2·7) | | 1·5 (-3·7, 6·7) | -1·7 (-6·0, 2·6) | | 1·7 (-4·0, 7·4) | -1·7 (-6·1, 2·7) | | 2·0 (-4·7, 8·8) | -1·8 (-6·4, 2·8) | |  |
| **5 mmol/L ≤ non-HDL-C** | 1·2 (-0·8, 3·3) | -0·9 (-2·6, 0·7) | | 0·5 (-0·4, 1·3) | -0·4 (-1·3, 0·4) | | 1·9 (-1·2, 4·9) | -1·3 (-3·7, 1·0) | | 1·1 (-0·8, 3·1) | -0·9 (-2·5, 0·8) | |  |
| **Thirds of years of education: lowest category** | 10·6 (-3·6, 24·9) | 8·3 (-0·7, 17·3) | | 5·2 (-2·2, 12·5) | 4·2 (-0·6, 9·1) | | 7·4 (-3·0, 17·8) | 5·9 (-0·8, 12·6) | | 11·8 (-3·9, 27·5) | 9·1 (-0·7, 18·8) | |  |
| **Thirds of years of education: middle category** | 3·7 (1·1, 6·2) | 3·8 (0·9, 6·7) | | 8·0 (2·7, 13·3) | 7·1 (1·9, 12·3) | | 5·7 (1·4, 10·0) | 5·3 (1·3, 9·3) | | 2·9 (0·7, 5·0) | 3·3 (0·6, 5·9) | |  |
| Men | | | | | | | | | | | | |  |
|  | **Cardiovascular** | | **Cancer** | **Cardiovascular** | | **Cancer** | **Cardiovascular** | | **Cancer** | **Cardiovascular** | | **Cancer** |  |
| **BMI < 20 kg/m^2^** | 3·2 (-9·5, 16·0) | | 10·3 (1·2, 19·4) | 0·7 (-2·5, 3·8) | | 2·2 (-0·9, 5·3) | 2·6 (-7·1, 12·4) | | 8·4 (0·5, 16·4) | 3·6 (-10·6, 17·8) | | 11·3 (1·4, 21·2) |  |
| **25 ≤ BMI < 30 kg/m^2^** | -1·3 (-4·2, 1·6) | | -0·4 (-2·0, 1·2) | -2·5 (-8·1, 3·1) | | -1·0 (-4·8, 2·8) | -1·7 (-5·7, 2·2) | | -0·5 (-2·7, 1·6) | -1·1 (-3·7, 1·5) | | -0·3 (-1·7, 1·1) |  |
| **30 kg/m^2^ ≤ BMI** | 0·4 (-1·3, 2·1) | | -0·2 (-1·6, 1·2) | 1·1 (-3·1, 5·4) | | -0·6 (-4·7, 3·4) | 1·1 (-3·3, 5·6) | | -0·7 (-4·8, 3·4) | 0·2 (-0·9, 1·3) | | -0·1 (-1·0, 0·7) |  |
| **120 ≤ SBP < 140 mmHg** | 2·4 (-5·3, 10·1) | | 3·5 (-1·6, 8·6) | 3·3 (-7·5, 14·2) | | 5·7 (-2·4, 13·8) | 3·3 (-6·9, 13·4) | | 5·0 (-2·0, 11·9) | 2·2 (-4·8, 9·2) | | 3·1 (-1·5, 7·7) |  |
| **140 ≤ SBP < 160 mmHg** | 4·6 (0·0, 9·1) | | 1·8 (-0·6, 4·1) | 7·6 (1·0, 14·2) | | 3·2 (-0·7, 7·1) | 8·2 (0·5, 16·0) | | 3·2 (-1·0, 7·4) | 3·7 (-0·4, 7·7) | | 1·4 (-0·6, 3·5) |  |
| **160 mmHg ≤ SBP** | 8·5 (2·9, 14·1) | | 2·1 (-0·6, 4·8) | 11·4 (5·4, 17·5) | | 2·6 (-0·7, 5·9) | 4·9 (0·9, 8·9) | | 1·2 (-0·4, 2·9) | 8·8 (2·2, 15·4) | | 2·2 (-0·8, 5·2) |  |
| **3 ≤ non-HDL-C < 4 mmol/L** | -3·7 (-8·9, 1·5) | | 2·7 (-4·8, 10·2) | -3·7 (-8·8, 1·5) | | 2·8 (-5·2, 10·8) | -2·5 (-5·9, 1·0) | | 1·7 (-3·4, 6·9) | -3·9 (-9·4, 1·6) | | 2·8 (-5·0, 10·7) |  |
| **4 ≤ non-HDL-C < 5 mmol/L** | -2·3 (-7·3, 2·6) | | -0·6 (-5·0, 3·7) | -1·5 (-4·5, 1·4) | | -0·5 (-3·5, 2·5) | -2·1 (-6·4, 2·1) | | -0·6 (-4·4, 3·2) | -2·4 (-7·8, 2·9) | | -0·6 (-5·2, 3·9) |  |
| **5 mmol/L ≤ non-HDL-C** | 1·1 (0·0, 2·2) | | -0·4 (-1·1, 0·4) | 2·1 (-0·4, 4·6) | | -0·4 (-1·2, 0·5) | 2·1 (-0·6, 4·8) | | -0·6 (-2·0, 0·8) | 0·8 (0·0, 1·7) | | -0·3 (-1·0, 0·4) |  |
| **Thirds of years of education: lowest category** | 10·8 (2·7, 19·0) | | 7·2 (-0·3, 14·7) | 10·4 (2·8, 17·9) | | 6·1 (-0·2, 12·5) | 6·9 (1·2, 12·6) | | 4·7 (-0·4, 9·8) | 11·5 (2·8, 20·3) | | 7·6 (-0·3, 15·6) |  |
| **Thirds of years of education: middle category** | 3·6 (0·7, 6·5) | | 0·2 (-3·5, 3·8) | 3·0 (0·4, 5·6) | | 0·3 (-3·0, 3·5) | 4·5 (0·8, 8·2) | | 0·3 (-4·5, 5·1) | 3·5 (0·5, 6·5) | | 0·2 (-3·4, 3·7) |  |

Population-attributable fractions for 20-year mortality risks at age 50 were estimated with the use of Weibull regression models. PAFs for single risk factors and aggregated PAF for the risk factors combined are shown. Abbreviations: SBP, systolic blood pressure; non-HDL-C, non-high-density lipoprotein cholesterol; BMI, body-mass index.

# References

1.	Böthig S. WHO MONICA Project: objectives and design. Int J Epidemiol. 1989;18(3 Suppl 1):S29-37. PubMed PMID: 2807705.

2.	Evans A, Salomaa V, Kulathinal S, Asplund K, Cambien F, Ferrario M, et al. MORGAM (an international pooling of cardiovascular cohorts). Int J Epidemiol. 2004 Aug 27;34(1):21–7. doi:10.1093/ije/dyh327

3.	Buuren SV, Groothuis-Oudshoorn K. **mice** : Multivariate Imputation by Chained Equations in *R*. J Stat Softw. 2011;45(3). doi:10.18637/jss.v045.i03

4.	White IR, Royston P. Imputing missing covariate values for the Cox model. Stat Med. 2009 Jul 10;28(15):1982–98. doi:10.1002/sim.3618 PubMed PMID: 19452569; PubMed Central PMCID: PMC2998703.

5.	Schemper M, Smith TL. A note on quantifying follow-up in studies of failure time. Control Clin Trials. 1996 Aug;17(4):343–6. doi:10.1016/0197-2456(96)00075-x PubMed PMID: 8889347.

6.	Korn EL, Graubard BI, Midthune D. Time-to-Event Analysis of Longitudinal Follow-up of a Survey: Choice of the Time-scale. Am J Epidemiol. 1997 Jan 1;145(1):72–80. doi:10.1093/oxfordjournals.aje.a009034

7.	Hjort NL. On Inference in Parametric Survival Data Models. Int Stat Rev Rev Int Stat. 1992 Dec;60(3):355. doi:10.2307/1403683

8.	Song L, Zhang S, Li H, Hansson O, Sonestedt E, Borné Y. Comparison of risk factors for Parkinson’s disease, coronary events and ischemic stroke. NPJ Park Dis. 2022 Aug 25;8(1):107. doi:10.1038/s41531-022-00374-z PubMed PMID: 36008425; PubMed Central PMCID: PMC9411547.

9.	Laaksonen MA, Virtala E, Knekt P, Oja H, Härkänen T. *SAS* Macros for Calculation of Population Attributable Fraction in a Cohort Study Design. J Stat Softw. 2011;43(7). doi:10.18637/jss.v043.i07

10.	Leong DP, Rangarajan S, Rosengren A, Oguz A, Alhabib KF, Poirier P, et al. Medications for blood pressure, blood glucose, lipids, and anti-thrombotic medications: relationship with cardiovascular disease and death in adults from 21 high-, middle-, and low-income countries with an elevated body mass index. Eur J Prev Cardiol. 2022 Oct 20;29(14):1817–26. doi:10.1093/eurjpc/zwac069 PubMed PMID: 35512128.

11.	Harrell FE. Regression Modeling Strategies: With Applications to Linear Models, Logistic Regression, and Survival Analysis [Internet]. New York, NY: Springer New York; 2001 [cited 2025 Feb 24]. (Springer Series in Statistics). Available from: http://link.springer.com/10.1007/978-1-4757-3462-1 doi:10.1007/978-1-4757-3462-1

12.	Lim SS, Vos T, Flaxman AD, Danaei G, Shibuya K, Adair-Rohani H, et al. A comparative risk assessment of burden of disease and injury attributable to 67 risk factors and risk factor clusters in 21 regions, 1990–2010: a systematic analysis for the Global Burden of Disease Study 2010. The Lancet. 2012 Dec;380(9859):2224–60. doi:10.1016/S0140-6736(12)61766-8

13.	Cheng S, Claggett B, Correia AW, Shah AM, Gupta DK, Skali H, et al. Temporal trends in the population attributable risk for cardiovascular disease: the Atherosclerosis Risk in Communities Study. Circulation. 2014 Sep 2;130(10):820–8. doi:10.1161/CIRCULATIONAHA.113.008506 PubMed PMID: 25210095; PubMed Central PMCID: PMC4161984.
